# Regional uptakes from early-frame amyloid PET and ^18^F-FDG PET scans are comparable independent of disease state

**DOI:** 10.1186/s41824-021-00123-0

**Published:** 2022-01-18

**Authors:** Alison Myoraku, Gregory Klein, Susan Landau, Duygu Tosun, for the Alzheimer’s Disease Neuroimaging Initiative

**Affiliations:** 1 Northern California Institute for Research and Education, VA Medical Center, 4150 Clement Street, 114M, San Francisco, CA 94121 USA; 2 Department of Psychiatry and Behavioral Sciences, Stanford University, Stanford, CA 94305 USA; 3 Roche Pharma Research and Early Development, Basel, Switzerland; 4 Helen Wills Neuroscience Institute, University of California, Berkeley, CA 94720-3190 USA; 5 Department of Radiology and Biomedical Imaging, University of California, San Francisco, San Francisco, CA 94143 USA

**Keywords:** Early-frame imaging, Amyloid, FDG, Alzheimer’s disease

## Abstract

**Purpose:**

Positron emission tomography (PET) imaging with amyloid-beta (Aβ) tracers and 2-[18F] fluoro-2-Deoxy-d-glucose (^18^F-FDG) is extensively employed in Alzheimer’s disease (AD) studies as biomarkers of AD pathology and neurodegeneration. To reduce cost and additional burdens to the patient, early-frame uptake during Aβ PET scanning has been proposed as a surrogate measure of regional glucose metabolism. Considering the disease state specific impact of AD on neurovascular coupling, we investigated to what extent the information captured in the early frames of an Aβ-PET (^18^F-florbetapir or ^18^F-florbetaben) scan is comparable to that of a ^18^F-FDG PET scan, independent of disease state.

**Method:**

A partial correlation was performed on early-frame ^18^F-florbetapir and ^18^F-FDG regional data from 100 participants. In a secondary analysis, we compared 92 ^18^F-florbetapir and 21 ^18^F-florbetaben early-frame Aβ scans from cognitively unimpaired and mild cognitive impairment participants to ascertain if regional early-frame information was similar across different Aβ-PET radioligands.

**Results:**

The partial correlation of early-frame ^18^F-florbetapir with ^18^F-FDG was significant in all 84 brain ROIs, with correlation values ranging from 0.61 to 0.94. There were no significant differences between early-frame ^18^F-florbetapir and ^18^F-florbetaben images.

**Conclusion:**

Overall, we find that the regional uptake measurements from early-frame ^18^F-florbetapir are strongly correlated with regional glucose metabolism as measured in ground-truth ^18^F-FDG PET scans, regardless of disease state. Future studies should focus on longitudinal early-frame amyloid PET imaging studies to further assess the value of early-frame imaging as a marker of brain metabolic decline.

**Supplementary Information:**

The online version contains supplementary material available at 10.1186/s41824-021-00123-0.

## Background

Alzheimer’s disease (AD), the most common form of dementia, is a debilitating neurodegenerative disease that affects approximately 14.5% of the American population (Rajan et al. 2019). AD is pathologically characterized by amyloid-beta (Aβ) plaques and tau pathology (neurofibrillary tangles) which can be detected with Aβ positron emission tomography (PET) scans and used to differentiate AD from other forms of dementia. These Aβ plaques can occur as many as 10–15 years prior to the onset of observable cognitive decline and are considered an important early biomarker for AD prevention (Rowe et al. 2010). Given the utility of Aβ PET imaging, there are now multiple radioligand tracers including ^11^C-labeled Pittsburgh Compound-B (C-PIB), ^18^F-florbetapir, and ^18^F-florbetaben that can be used for in vivo imaging (Klunk et al. 2004; Wong et al. 2010; Villemagne et al. 2011).

However, quantification of Aβ burden alone is not always sufficient in the diagnosis of AD and it is important to consider additional aspects of pathophysiology, including neurodegeneration which could be reflected as early changes in cerebral blood flow (perfusion) and glucose metabolism (Jack et al. 2018; Ahmad et al. 2020). Studies show that differences in perfusion exist between healthy controls and AD patients and may also be present long before clinical symptoms of AD are observed (Hays et al. 2016; Kisler et al. 2017). Many have shown the importance of evaluating glucose metabolism in the brains of patients at risk of or diagnosed with AD (Chen and Zhong 2013; Gaitán et al. 2019). In particular, hypometabolism in the precuneus, posterior cingulate, and hippocampus has been observed in AD consistently, and associated with changes in synaptic density and correlated with cognitive impairment (Friedland et al. 1983; Maldjian and Whitlow 2012; Minoshima et al. 1997; Calsolaro and Edison 2016).

Acquiring information on all these aspects of the disease currently requires multiple PET scans, which is expensive and exposes the patient to multiple doses of radiation. As a solution to this problem, researchers have started to investigate the degree to which the early frames of an amyloid scan can capture the information provided by PET scans administered to assess glucose metabolism.

The early-frames of an amyloid scan provide information akin to perfusion, as the amyloid-binding tracer perfuses through various regions throughout the brain (Blomquist et al. 2008). This information, in turn, may be used as a measure of metabolism, given that vascular and metabolic coupling has been established in cognitively normal older adults and those with degenerative dementias (Silverman 2004). Current literature on the topic suggests that the agreement between amyloid scans and F-18 fluorodeoxyglucose (^18^F-FDG) scans is promising (Hsiao et al. 2012; Tiepolt et al. 2016). Specifically, in one study with 33 subjects [11 mild cognitive impairment (MCI), 22 dementia (11 AD)] comparing ^18^F-florbetaben amyloid scans with ^18^F-FDG scans, researchers reported highly significant correlations (ranging from 0.60 to 0.92), irrespective of the early-frame number or the brain region (Daerr et al. 2017). Another study with 52 MCI subjects compared both early frames and the relative rate of delivery (R1) of C-PIB PET scans to ^18^F-FDG scans and found high correlations between early-frame C-PIB and ^18^F-FDG (0.83 for frames 0–6 min and 0.4 for frames 1–8 min) (Oliveira et al. 2018). Although there is growing evidence for the role of early-frame amyloid images in differentiating between disease state or for detecting brain metabolism, the question of whether early-frame amyloid measures could be a surrogate marker of brain metabolism robustly across different disease states (given that the degree of neurovascular coupling might differ by disease state) remains to be answered. If true, this finding could be extremely beneficial in the context of a clinical trial that recruits across the AD spectrum and uses glucose metabolism as a measure of either treatment efficacy or illness progression. Furthermore, comparing different types of amyloid tracers (e.g. ^18^F-florbetaben vs ^18^F-florbetapir) and identifying their similarities and/or differences would allow for a greater understanding of influence of radioligand choice and potential interchangeability of early-frame amyloid scans in replacement of ^18^F-FDG PET scans.

The primary objective of this study was to assess the degree to which early-frame ^18^F-florbetapir scans capture information about glucose metabolism in ground-truth ^18^F-FDG scans in an observational cohort of individuals with and without cognitive impairment and dementia. Specifically, we assessed standardized uptake value ratio (SUVR) from early-frame ^18^F-florbetapir PET and ^18^F-FDG PET scans in 82 cerebral regions of interest and 2 two composite regions (see Additional file 1: Supplementary Table S6) and conducted partial correlations to assess the relationship between the two scan types and tested if these associations were modulated by clinical disease state. As a secondary objective, we compared ^18^F-florbetapir early-frame regional SUVR values with ^18^F-florbetaben early-frame SUVR values of non-demented individuals to assess the similarity of the information captured by each tracer type and ascertain if ^18^F-FDG PET scans can be replaced by either type of early-frame amyloid scan.

## Methods

### Study design

Data were obtained from the database of the Alzheimer’s Disease Neuroimaging Initiative (ADNI) (adni.loni.usc.edu). The National Institute of Aging, the National Institute of Biomedical Imaging and Bioengineering, the Food and Drug Administration, private pharmaceutical companies, and nonprofit organizations launched ADNI in 2004 as a public–private partnership. ADNI is a longitudinal multi-center natural history study designed to characterize clinical, neuropsychological, MRI and PET imaging, genetic, and biochemical biomarkers for early detection and tracking of AD (Jack et al. 2008). The principal investigator of ADNI is Michael Weiner, MD, VA Medical Center and University of California, San Francisco. For current information on ADNI, see www.adni-info.org.

### Study participants

Subjects of the main study were ADNI participants (CU, MCI, and Dementia) who underwent both early-frame ^18^F-florbetapir PET and ^18^F-FDG PET. Exclusion criteria for ADNI participants included any significant neurologic disease, such as Parkinson’s disease, multi-infarct dementia, Huntington’s disease, normal pressure hydrocephalus, progression supranuclear palsy, seizure disorder, subdural hematoma, multiple sclerosis, or history of significant head trauma followed by persistent neurologic deficits or known structural brain abnormalities. The time between the two scans was limited to less than 2.5 years. The cohort for the secondary objective was based on ADNI participants (CU and MCI only) who underwent early-frame ^18^F-florbetaben PET imaging augmented with age, sex, and diagnoses matched participants with early-frame ^18^F-florbetapir PET imaging. Selection was made a priori from all ADNI subjects based on the availability of complete data including cross-sectional imaging and longitudinal clinical and cognitive measures as of April 20th, 2021.

### PET acquisition

#### ^18^F-FDG PET

Participants were injected with 185 mBq (5 mCi) of ^18^F-FDG and a dynamic 3D scan of 6 5-min frames were acquired 30–60 min post injection. For each individual, an average of all frames was used in this analysis. Dynamic images were used as static images were not available.

#### Early-frame ^18^F-florbetapir/^18^F-florbetaben PET

Participants were injected with 370 mBq (10 mCi) of ^18^F-florbetapir or 300 mBq (8.1 mCi) of ^18^F-florbetaben with simultaneous initiation of the dynamic PET scan. The scan lasted 20 min and comprised the following sequence (4 × 15 s, 4 × 30 s, 3 × 60 s, 3 × 120 s, 2 × 240 s). An average of frames 4–11, i.e., average of 45 s to 6 min, as recommended by multiple studies were used in this analysis (Rostomian et al. 2011; Rodriguez-Vieitez et al. 2017).

### MRI acquisition

3T multimodality MRI data included a 3D MP-RAGE or IR-SPGR T1-weighted (T1w) MRI, as described online (http://adni.loni.usc.edu/methods/documents/mri-protocols).

### Image quality assurance

All images were visualized with MRIcron to check for outstanding artifacts and the overall quality of the acquisition.

### Image processing

A fully automated processing pipeline, Advanced Normalization Tools (ANTs) cortical thickness pipeline, was applied to each T1w scan and spatially normalized each image to a widely used T1w MRI template in stereotaxic space, the Montreal Neurological Institute/International Consortium for Brain Mapping (MNI-152) (Avants et al. 2011). Each anatomical MRI was segmented into *N* = 75 cortical and *N* = 7 subcortical regions-of-interest (ROIs) based on a previously published volumetric Hammers atlas (Hammers et al. 2003; Gousias et al. 2008).

Early-frame amyloid PET and ^18^F-FDG PET images were level 4 ADNI preprocessed data that had been smoothed to standard 8 FWHM, co-registered, standardized to a baseline image, averaged, and normalized (http://adni.loni.usc.edu/methods/pet-analysis-method/pet-analysis/). The averaged images were then rigid-aligned to corresponding T1w images, then spatially normalized to template space using the subject specific T1w image space to MNI space spatial normalization transformation. Standard uptake values for 82 regions of interest were then extracted from the registered PET images. The brainstem served as a reference region for both the early-frame ^18^F-florbetapir images and ^18^F-FDG images to estimate the regional standard uptake value ratio (SUVR) (Verger et al. 2021). Further, composite regions of interest comprising the average of the middle/inferior temporal, posterior cingulate cortex, and inferolateral remainder of the parietal lobe regions by hemisphere, as previously described in Landau et al. (2011), were also included.

### Statistical analyses

All analyses were conducted with statistical software in R (R Core Team 2017). All ROI-based analyses were adjusted for multiple comparisons with the False Discovery Rate (FDR) method.

#### Comparison of early-frame ^18^F-florbetapir and ^18^F-FDG

A partial correlation between regional early-frame ^18^F-florbetapir and regional ^18^F-FDG SUVR was performed with time between scans as a covariate. Additionally, Bland–Altman plots were used to show graphically the agreement between the two modalities across all regions. To assess the effect of disease state (i.e., CU, MCI, Dementia) on the inter-modality (i.e., early-frame ^18^F-florbetapir versus ^18^F-FDG) associations, a linear regression analysis between regional early-frame ^18^F-florbetapir and ^18^F-FDG uptakes was performed with clinical disease state as an interaction term and time between scans as a covariate. The linear regression analysis was repeated with the clinical dementia rating (CDR) sum of boxes score (continuous) in lieu of disease state (categorical) to assess if the association between early-frame 18F-florbetapir and ^18^F-FDG PET regional SUVR is modulated by the symptom severity. Additionally, bias between the two modalities was calculated as the difference between the ^18^F-florbetapir mean and the ^18^F-FDG mean, divided by the ^18^F-FDG mean (ground truth for glucose metabolism), multiplied by 100.

#### Comparison of early-frame ^18^F-florbetapir and ^18^F-florbetaben

We first compared the whole brain early-frame ^18^F-florbetapir and early-frame ^18^F-florbetaben patterns by taking the SUVRs of 82 cerebral regions per subject as vectors and computing cosine similarities between every pair of vectors. Linear regressions by region with covariates of age, diagnosis, and CDR sum of boxes score between early-frame ^18^F-florbetapir and early-frame ^18^F-florbetaben SUVR values were also conducted.

## Results

### Patient characteristics

#### Early-frame ^18^F-florbetapir versus ^18^F-FDG

**Fig. 1:**
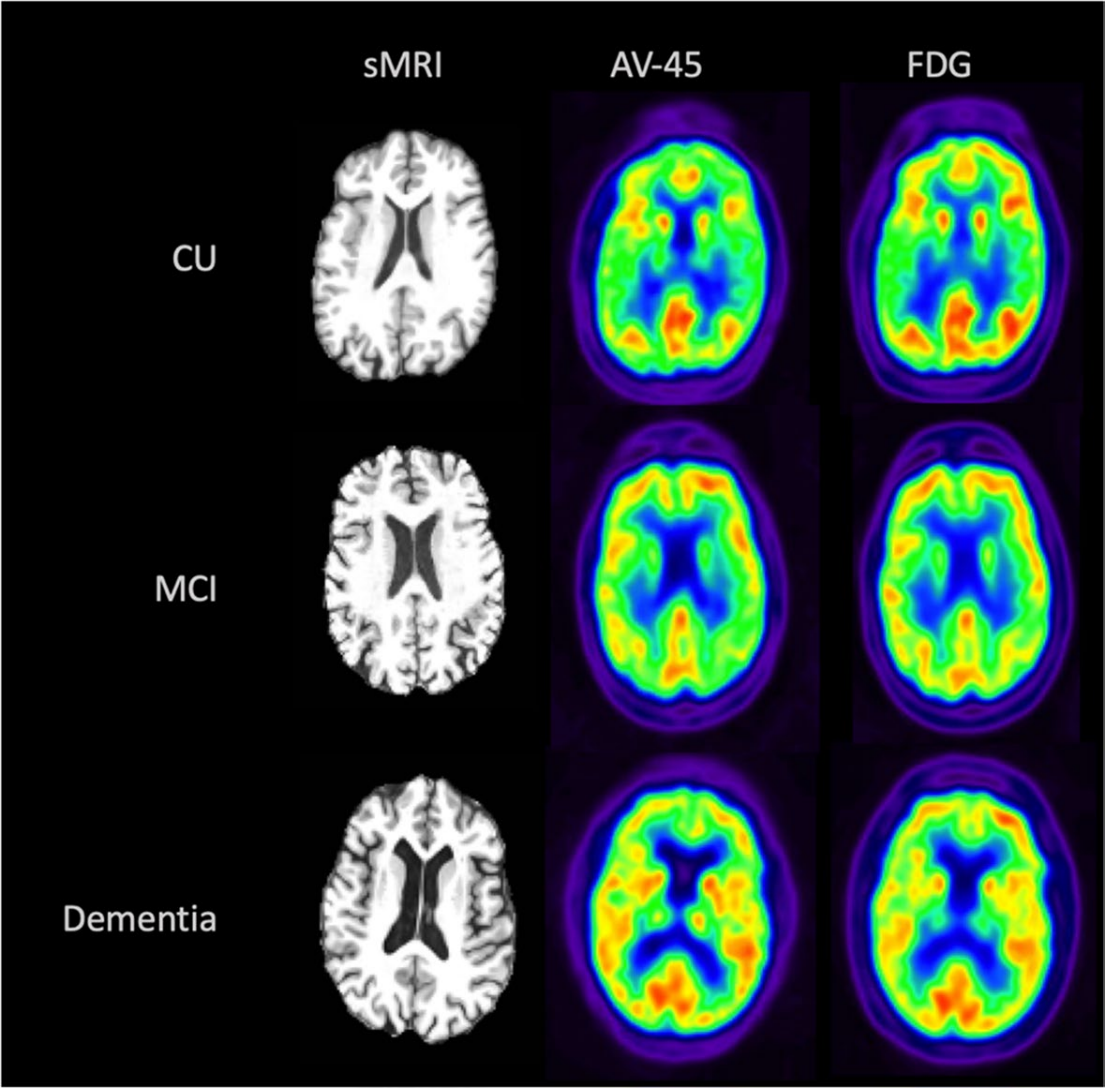
Visual comparison of sMRI, early-frame 18F-florbetapir, and ^18^F-FDG PET for participants from each disease state (CU, MCI, dementia)

Out of 103 cases with early-frame ^18^F-florbetapir and ^18^F-FDG PET scans available as of April 20, 2021, three cases (two CU and one MCI) were removed due to a T1 to PET registration error. Based on the clinical assessment done closest in time to the early-frame ^18^F-florbetapir PET visit, the final study cohort was composed of 52 CU individuals, 33 individuals with MCI, and 15 individuals with dementia. Demographic and clinical characteristics of these participants are displayed in Table 1 and sample images of structural MRI, early-frame ^18^F-florbetapir and ^18^F-FDG PET scans are available in Fig. 1.

**Table 1:** Characteristics of the cohort assessed for inter-modality associations between early-frame AV45-PET and FDG-PET regional SUVRs

Demographic	Cognitively unimpaired	Mild cognitive impairment	Dementia
*N*	52	33	15
Sex (*N* female, % female)	30 (57.7%)	13 (39.4%)	7 (46.7%)
Age (mean, SD)	74.42 (6.56)	82.10 (9.71)	73.90 (7.22)
Education, years (mean, SD)	16.46 (2.95)	16.22 (3.01)	14.87 (3.00)
Time between scans in years (mean, SD)	0.08 (0.44)	0.09 (0.46)	0.003 (0.01)
CDR-SB (mean, SD)	0.30 (0.94)	1.67 (1.62)	4.83 (2.93)

#### Early-frame ^18^F-florbetapir versus ^18^F-florbetaben

Out of 144 cases (122 ^18^F-florbetapir, 22 ^18^F-florbetaben) available as of April 20, 2021, one participant with an ^18^F-florbetaben scan was removed following an outlier detection criterion of 3 absolute *z*-scores from the mean of the distribution from patients (divided by diagnosis) for each ROI. An additional 30 ^18^F-florbetapir cases were removed to ensure that the mean age between groups was not statistically significantly different. Based on the clinical assessment done closest in time to the early-frame ^18^F-florbetapir PET visit, the final cohort for this objective was composed of 77 CU individuals and 36 individuals with MCI. This cohort was limited to CU and MCI due to an insufficient number of available AD cases (*N* = 2) with early-frame ^18^F-florbetaben scan. Demographic and clinical characteristics of these are displayed in Table 2.

**Table 2:** Characteristics of the cohort assessed early-frame 18F-florbetapir and early-frame 18F-florbetaben regional SUVR associations

Demographic	18F-florbetapir	18F-florbetaben	Comparison
*N*	92	21	
Sex (*N* female, % female)	49 (53.2%)	11 (52.4%)	*χ*^2^: 7.8e−31, *p*: 1.0
Age (mean, SD)	71.9	69.4 (5.4)	*t*: 1.96, *p*: 0.06
CDR-SB (mean, SD)	0.68	0.68 (1.4)	*t*: 0.04, *p*: 0.97
*Diagnosis*			
MCI	28	8	*χ*^2^: 0.02, *p*: 0.88
CU	64	13	

### Association between early-frame ^18^F-florbetapir and FDG regional SUVRs

**Fig. 2:**
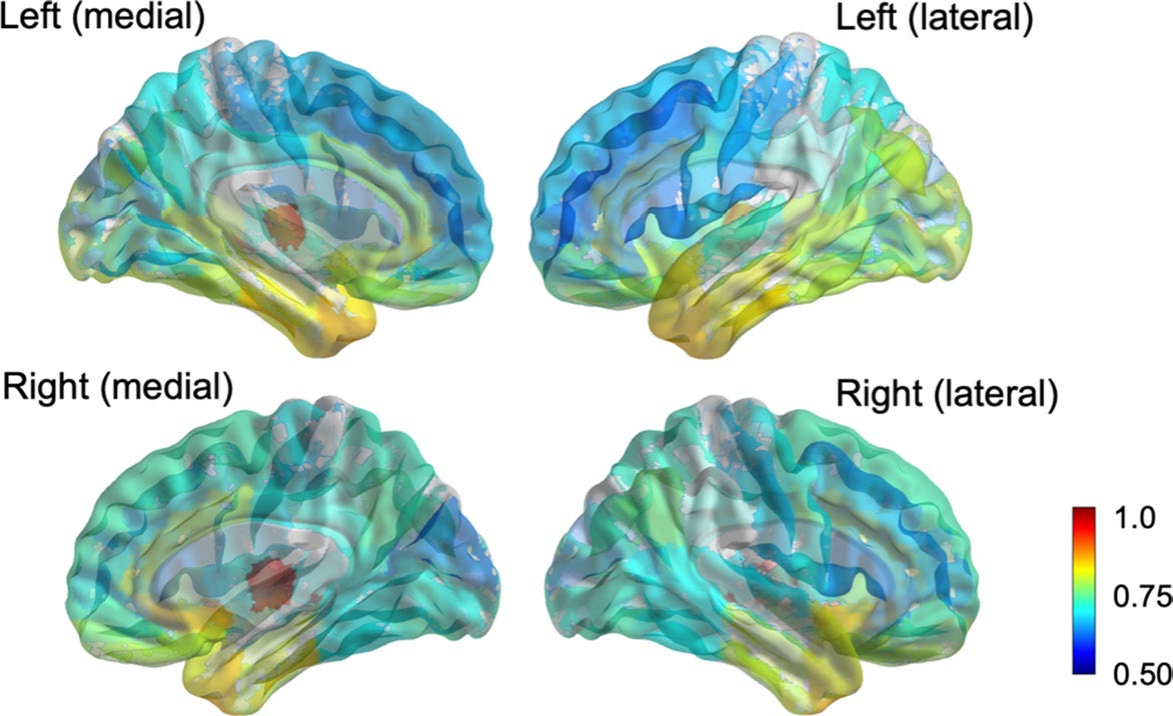
Brain maps of *R* values from partial correlation between early-frame 18F-florbetapir and ^18^F-FDG PET regional uptake

**Fig. 3:**
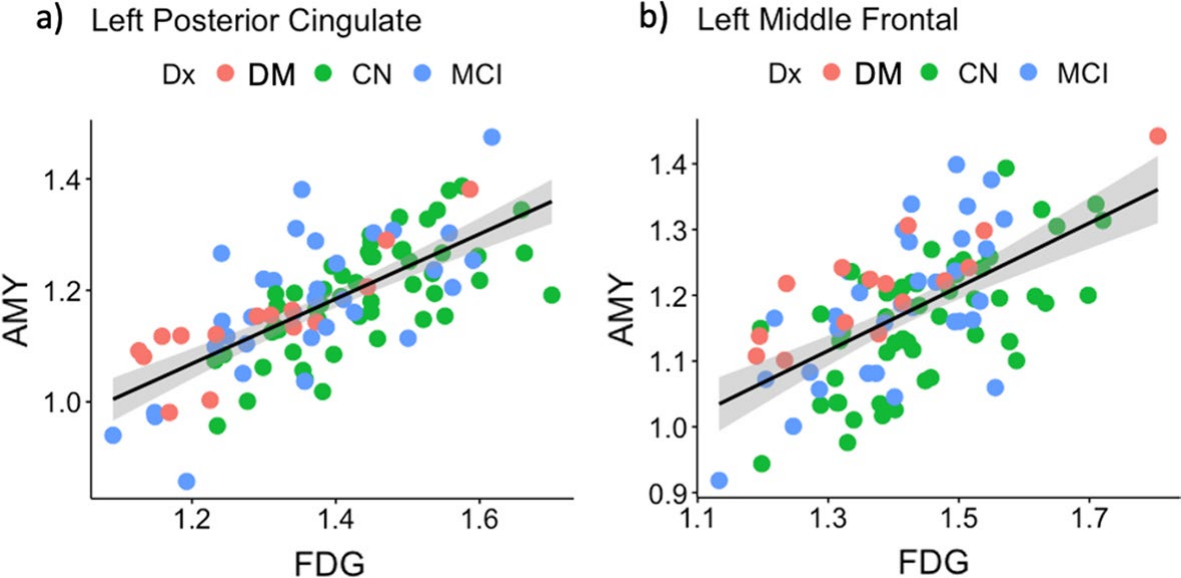
Comparison of the partial correlations between early-frame ^18^F-florbetapir and ^18^F-FDG PET regional uptake of **a** the left posterior cingulate gyrus (*r* = 0.70) and **b** the left middle frontal gyrus (*r* = 0.61). *DM* dementia, *MCI* mild cognitive impairment, *CU* cognitively unimpaired, *AMY* amyloid PET SUVR

**Fig. 4:**
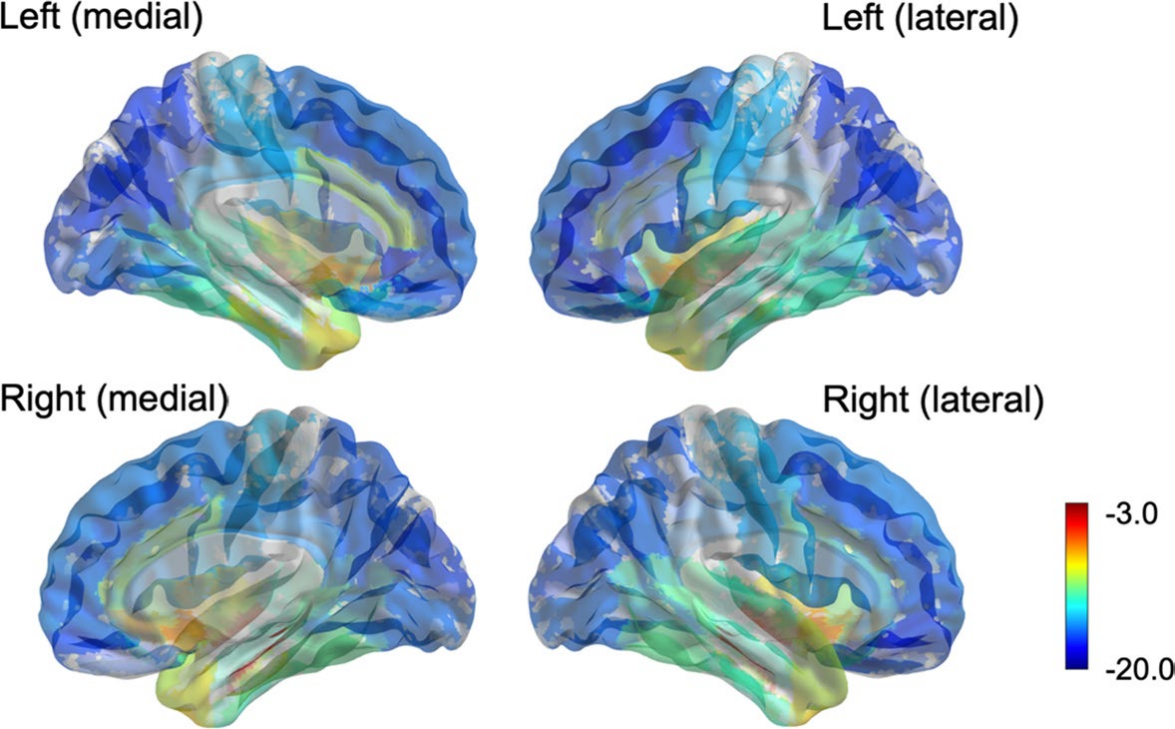
Bias Maps of early-frame 18F-florbetapir versus F-FDG. Bias was calculated as (early-frame 18F-florbetapir mean—^18^F-FDG mean)/^18^F-FDG mean) × 100

**Fig. 5:**
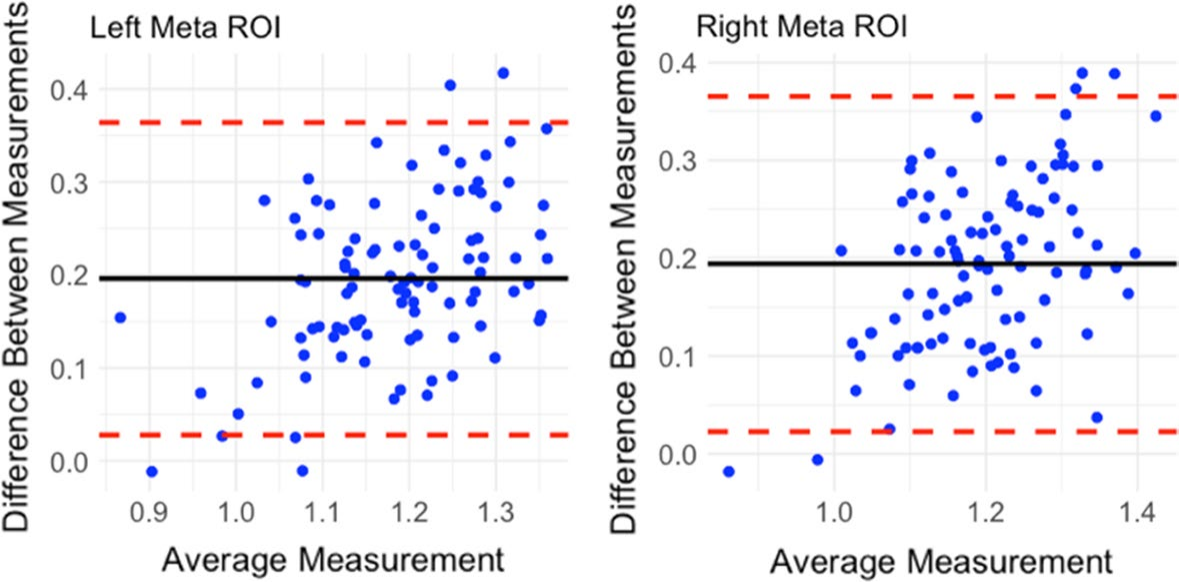
Bland–Altman comparison of regional ^18^F-florbetapir and regional ^18^F-FDG SUVR for left and right meta-ROI. Mean difference: 0.15 (CI − 0.006 to 0.31). Range of average of differences: − 0.006 (right thalamus) to 0.27 (right cuneus)

The partial correlation between early-frame ^18^F-florbetapir and ^18^F-FDG PET SUVRs was significant in all 82 cerebral regions and 2 composite regions, ranging from 0.61 to 0.94 (mean = 0.74) as displayed in Fig. 2. The highest correlation between the early-frame ^18^F-florbetapir and ^18^F-FDG data within cortical regions was in the anterior temporal lobe (left: *r* = 0.85, *p* < 2e−16; right: *r* = 0.85, *p* < 2e−16). Figure 3 provides a graphical comparison of the left posterior cingulate gyrus (*r* = 0.70), a region often observed in clinical trials, and the left middle frontal gyrus (*r* = 0.61), which exhibited the lowest partial correlation. The average ^18^F-FDG SUVR for the whole brain was 1.21 while the average early-frame ^18^F-florbetapir SUVR was 1.07. Figure 4 visualizes the measurement bias between early-frame ^18^F-florbetapir and ^18^F-FDG PET when using F-FDG as the gold-standard (also reported in Additional file 1: Supplementary Table S1). As displayed in the figure, there is a regional variance such that the difference between early-frame ^18^F-florbetapir SUVR and ^18^F-FDG SUVR is greater in frontoparietal regions compared to temporal regions. Partial correlations for all 82 cerebral regions and 2 composite regions are reported in Additional file 1: Supplementary Table S2. The Bland–Altman plots demonstrated that regional early-frame 18F-florbetapir and ^18^F-FDG SUVR are quite concordant, largely showing no change in residual variance with increasing ^18^F-FDG SUVR (Fig. 5, Additional file 1: Supplementary Table S3, Additional file 1: Supplementary Fig. S1).

### Effect of clinical disease stage on the associations between early-frame ^18^F-florbetapir and FDG regional SUVRs

Compared to CU participants, the early-frame ^18^F-florbetapir versus ^18^F-FDG uptake association in MCI and Dementia participants was significantly stronger only in the right thalamus (MCI: *β* = 0.37, *p* = 0.016; Dementia: *β* = 0.37, *p* = 0.025). In all other regions, clinical disease stage did not have a significant effect on the association. In the linear regression analysis accounting for disease stage differences by including CDR-SB instead of categorical clinical diagnosis as a linear interaction term the CDR sum of boxes score did not have statistically significant (*p* < 0.05 FDR corrected) modulation effect on the association between early-frame ^18^F-florbetapir and ^18^F-FDG uptake. This second linear regression analysis did however reiterate a significant correlation between ^18^F-florbetapir and ^18^F-FDG uptake (Additional file 1: Supplementary Table S4).

### Association between early-frame ^18^F-florbetapir and early-frame ^18^F-florbetaben regional SUVRs

**Fig. 6:**
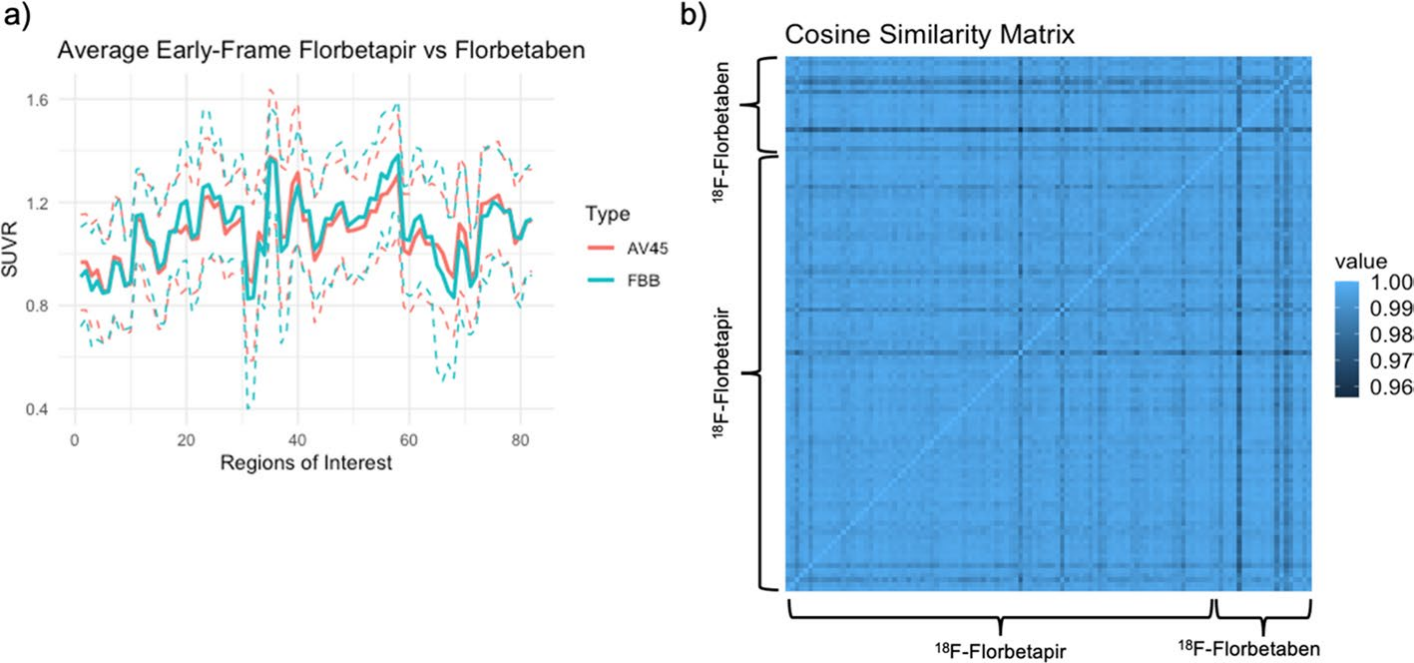
Comparisons of early-frame ^18^F-florbetapir and ^18^F-florbetaben Whole Brain SUVR Profile. **a**) Regional averages are displayed as a continuous line to help visualize a whole brain SUVR profile for each tracer and to easily compare between the early-frame ^18^F-florbetapir and early-frame ^18^F-florbetaben regional SUVRs. **b**) A cosine similarity matrix between all early-frame 18F-florbetapir and 18F-florbetaben vector pairs. A table with anatomical regions corresponding to the numbers in this graph is included in the Supplementary Material (Additional file 1: Supplementary Table S6). AV45 = ^18^F-florebetapir, FBB = ^18^F-florbetaben

Figure 6a displays the average early-frame ^18^F-florbetapir brain profile with the average early-frame ^18^F-florbetaben profile with confidence bounds calculated as two standard deviations from the mean. Regional averages are displayed as a continuous line to help visualize a SUVR brain profile for each tracer and to easily compare between the two. As displayed in the cosine similarity matrix in Fig. 6b, similarities between and within tracer type were largely the same, ranging from 0.96 to 1.00 in this example. A visual comparison of the magnitude of similarity between and within tracer type can be found in Additional file 1: Supplementary Fig. S2. Given the large overlap between these two groups and the high similarity measures, we find that the two tracers did not differ statistically in the relative regional information collected. The results of the linear regression by region (covariates: age, diagnosis, and CDR sum of boxes score) between early-frame ^18^F-florbetapir and ^18^F-florbetaben scans can be found in Additional file 1: Supplementary Table S5.

## Discussion

In this study, we compared early-frame ^18^F-florbetapir PET and ^18^F-FDG PET scans from 100 cognitively diverse (CU, MCI, and Dementia) participants in 84 different cerebral regions-of-interest to assess the degree to which early-frame amyloid scans can capture information about cerebral glucose metabolism typically measured by ^18^F-FDG PET scans. We found that the correlation between early-frame ^18^F-florbetapir scans and ^18^F-FDG scans was moderate to strong across all ROIs and disease states, with the exception of the right thalamus where the strength of the inter-modality association was modulated in MCI and Dementia participants relative to CU. The strength of the correlation was also confirmed with a separate analysis that used CDR-SB scores instead of disease state, and also found no significant effect of score on the association between early-frame amyloid and ^18^F-FDG. Another major finding of our analyses was that early-frame ^18^F-florbetapir and ^18^F-florbetaben regional SUVRs do not significantly differ. Taken together, these results suggest that regional SUVR from early-frame amyloid scans, regardless of the tracer used, is well-correlated with regional SUVR from ^18^F-FDG PET scans and could potentially be used for diagnostic purposes in individuals along the AD spectrum.

The first major finding that the correlation between early-frame ^18^F-florbetapir and ^18^F-FDG regional SUVRs is significant and moderate to high (i.e., between 0.61 and 0.94) is consistent with previous studies that investigated a similar question using different amyloid tracers (i.e., C-PIB) or the addition of the R1 measure from amyloid scans (Daerr et al. 2017; Oliveira et al. 2018; Rostomian et al. 2011). Our finding is also of importance because it validates this correlation in a sample size roughly 2–3 times larger than in previous studies. Furthermore, we included a composite of temporoparietal regions that would be likely examined in an AD treatment trial and found the partial correlation between early-frame ^18^F-florbetapir and ^18^F-FDG was moderate and significant. Our observation that these inter-modality correlations are moderate to strong regardless of disease state also has important implications for the potential replacement of ^18^F-FDG scans with early-frame amyloid scans for more efficient study designs without loss of sensitivity. Specifically, it suggests that early-frame amyloid scans could be used as a surrogate measure to help quantify glucose metabolism in participants across the cognitive spectrum without concern of the correlation fluctuating between diagnoses. While this would be particularly beneficial for Alzheimer’s clinical trials that recruit participants with a wide range of clinical stages and participants that might convert between different disease states during the trial, we believe that these results should be replicated in a larger, more balanced cohort prior to making these claims.

Differences between disease states in the right thalamus were subtle—the correlation between early frames and ^18^F-FDG PET was positive for all three disease states, with dementia and MCI groups exhibiting slightly stronger (more positive) correlations. However, there is no biological reason to expect this region to vary by disease state and this finding needs to be replicated in future datasets before any conclusions can be drawn from this observation.

While partial correlations between early-frame ^18^F-florbetapir and ^18^F-FDG PET SUVR were relatively strong in temporal regions and some central structures, we observed that the strength of the correlations was weaker in some frontal and occipital regions (Fig. 4 and Additional file 1: Supplementary Table S1). Specifically, we see that ^18^F-FDG SUVR tends to be higher than early-frame ^18^F-florbetapir SUVR in these regions. This is not an unexpected result, as we know that the coupling between perfusion and glucose metabolism is not always consistent throughout the brain (Tosun et al. 2016). For example, hypoperfusion of frontal regions in patients with AD has been previously reported (Kataoka et al. 2010; Thomas et al. 2015), while hypometabolism is not consistently found in these regions (Kato et al. 2016). Lower correlations between the two modalities in the occipital lobe were also reported by Hsiao et al. (2012). We acknowledge that while the two imaging modalities are not identical, they are similar enough (as evidenced by the correlations in Additional file 1: Supplementary Table S2) for certain applications, such as patient diagnosis and detection of treatment effect.

As a second major finding, we observed that within early frame amyloid scans, tracer type has no significant effect on standard uptake values in CU and MCI patients. With cosine similarity measures between and within tracer type ranging from 0.96 to 1.00, we believe that replacing one tracer with the other will not affect clinical decisions. By extension, these findings indicate that ^18^F-florbetapir and ^18^F-florbetaben early-frame amyloid scans could replace ^18^F-FDG scans in the measurement of cerebral glucose metabolism. Given the relatively small sample size of ^18^F-florbetaben scans (*N* = 21), it would be beneficial to replicate this analysis in a larger cohort. Additionally, it would be important to ascertain if early-frame ^18^F-florbetapir and ^18^F-florbetaben data is comparable in patients with advanced cognitive impairment (i.e., dementia).

There are a few limitations to this study worth mentioning. First, there are multiple components of an early-frame amyloid scan that can be considered for comparison with ^18^F-FDG. Many current studies suggest that early-frame amyloid and ^18^F-FDG are the most comparable when using the R1 measure of early frame scans, which represents the relative delivery rate of the tracer. In this analysis, we chose to focus on a simpler comparison with ^18^F-FDG that did not require kinetic modeling which yielded robust correlations between early-frame amyloid and ^18^F-FDG in this study. Additionally, the role of blood perfusion in this association between early-frame amyloid and ^18^F-FDG PET scans should be assessed in future studies as more arterial spin labeled (ASL) MRI data becomes available in early-frame studies. Second, we recognize that the early-frame ^18^F-florbetapir and ^18^F-florbetaben comparison would have been more complete with a partial correlation analysis between SUVRs from each early-frame image and a corresponding ^18^F-FDG image. However, ^18^F-FDG scans were not available for many ADNI participants selected for the early-frame ^18^F-florbetaben study. Comparing the partial correlation coefficients of early-frame ^18^F-florbetapir and ^18^F-FDG to those of ^18^F-florbetaben and ^18^F-FDG would be a worthwhile future analysis to further expand on the findings in this study.

We should note that the current study is based on a convenience cohort where the degree of true population representation is not known. Most notable, due to strict exclusion of participants with vascular pathology etiologies in ADNI studies, vascular disease burden was overall low in our study cohort compared to the general population. Although strong evidence suggests that vascular disease influences glucose metabolism and regional cerebral perfusion, the degree to which cerebral perfusion and glucose metabolism correlate in the general population might impact the generalizability of findings in this study (Pascual et al. 2010; Leuzy et al. 2018; Yan et al. 2018). Furthermore, we recognize the limitation of exploring this correlation in a cross-sectional cohort. A very important aspect of investigating disease pathophysiology is to track its progression over time, which can only be accomplished through a longitudinal data set. Ideally, it would be important to identify whether changes in glucose metabolism over time could be detected in early-frame amyloid scans as they are in ^18^F-FDG scans. Put more simply, does this correlation between early-frame amyloid and ^18^F-FDG scans remain significant over time? The finding from this study that the correlation between early-frame amyloid and ^18^F-FDG scans is robust across disease states may indicate that the correlation would be preserved over the course of the disease, although this would need to be confirmed in a longitudinal dataset. Lastly, we acknowledge that our findings should be taken cautiously given the small number of individuals with dementia included in this analysis (15 out of 100).

## Conclusion

Here, we showed that within a diverse cohort of individuals (cognitively unimpaired, have mild cognitive impairment, or dementia), early-frame ^18^F-florbetapir and ^18^F-FDG PET regional SUVRs are moderately to strongly correlated. Overall, this study will likely have significant implications for researchers studying biomarkers related to glucose metabolism in Alzheimer’s disease in a cohort of participants with a wide range of clinical stages, especially for those who may convert between disease states during the study period.

## Supplementary Information

**Additional file 1.** Supplementary Information.

## Data Availability

The datasets generated during and/or analyzed during the current study are not publicly available due to rules set forth by the Alzheimer’s Disease Neuroimaging Initiative, but are available from the corresponding author on reasonable request.
