# Supplementary material for: Regional uptakes from early-frame amyloid PET and 18F-FDG PET scans are comparable independent of disease state
Source: Eur J Hybrid Imaging. 2022 Jan 18;6:2. doi: 10.1186/s41824-021-00123-0 (PMC8763988; doi:10.1186/s41824-021-00123-0)

**Supplemental Material**

**Supplementary Table 1: Bias between early-frame ^18^F-florbetapir and ^18^F-FDG PET.**Calculated as ((early-frame ^18^F-florbetapir - ^18^F-FDG) / ^18^F-FDG) x 100.

|  | **Left** | **Right** |  | **Left** | **Right** |
| --- | --- | --- | --- | --- | --- |
| **ROI** | Bias (%) | Bias (%) | **ROI** | Bias (%) | Bias (%) |
| ***Temporal Lobe*** |  |  | ***Insula and Cingulate*** |  |  |
| Hippocampus | -4.47 | -4.97 | Posterior long gyrus (insula) | -6.49 | -6.26 |
| Amygdala | -2.88 | -3.26 | Anterior long gyrus (insula) | -9.34 | -9.38 |
| Anterior Temporal (M) | -8.78 | -8.62 | Anterior short gyrus (insula) | -11.80 | -11.40 |
| Anterior Temporal (L) | -11.04 | -10.73 | Middle short gyrus (insula) | -9.09 | -8.14 |
| Parahippocampal | -9.69 | -10.34 | Posterior short gyrus (insula) | -8.96 | -8.98 |
| Middle/Inferior Temp | -13.22 | -12.76 | Anterior inferior cortex (insula) | -7.64 | -7.20 |
| Fusiform | -10.76 | -11.40 | ACC | -10.40 | -10.71 |
| Posterior Temp | -12.67 | -12.04 | PCC | -15.18 | -15.66 |
| Superior Temp (A) | -11.14 | -10.50 | ***Occipital Lobe*** |  |  |
| Superior Temp (P) | -12.91 | -12.71 | Lingual gyrus | -16.00 | -16.10 |
| ***Frontal Lobe*** |  |  | Cuneus | -17.29 | -17.17 |
| Middle Frontal | -17.46 | -16.99 | Lateral occipital | -16.83 | -16.21 |
| Precentral gyrus | -14.79 | -14.66 | ***Parietal Lobe*** |  |  |
| Straight | -14.98 | -14.24 | Postcentral gyrus | -14.96 | -14.98 |
| Anterior Orbital | -16.59 | -17.32 | Superior Parietal | -17.13 | -16.53 |
| Inferior Frontal | -16.62 | -15.65 | Inferolateral Remainder | -17.10 | -16.15 |
| Superior Frontal | -16.00 | -15.75 | ***Central Structures*** |  |  |
| Medial Orbital | -16.65 | -16.58 | Caudate | -16.10 | -16.96 |
| Lateral Orbital | -18.20 | -17.57 | Nucleus Accumbens | -7.20 | -7.01 |
| Posterior Orbital | -13.45 | -12.85 | Putamen | -11.10 | -11.47 |
| Subgenual Frontal | -7.64 | -7.60 | Thalamus | 0.29 | 0.69 |
| Subcallosal | -7.72 | -7.35 | Pallidum | -6.72 | -10.08 |
| Pre-subgenual | -8.81 | -8.05 | ***Composite ROI*** | -14.84 | -15.38 |

**Supplementary Table 2: Partial Correlations for Early-frame Amyloid and F-FDG Comparison**

|  | Left | | | Right | | |
| --- | --- | --- | --- | --- | --- | --- |
| ROI | AV-45 ± SD | FDG ± SD | R | AV-45 ± SD | FDG ± SD | R |
| *Temporal Lobe* |  |  |  |  |  |  |
| Hippocampus | 0.94 ± 0.11 | 0.98 ± 0.1 | 0.81*** | 0.94 ± 0.1 | 0.99 ± 0.1 | 0.80*** |
| Amygdala | 0.91 ± 0.12 | 0.94 ± 0.1 | 0.80*** | 0.89 ± 0.1 | 0.92 ± 0.1 | 0.79*** |
| Anterior Temporal (M) | 0.83 ± 0.12 | 0.91 ± 0.12 | 0.85*** | 0.83 ± 0.11 | 0.91 ± 0.11 | 0.85*** |
| Anterior Temporal (L) | 1.08 ± 0.13 | 0.96 ± 0.13 | 0.83*** | 0.97 ± 0.12 | 1.08 ± 0.13 | 0.80*** |
| Parahippocampal | 0.85 ± 0.1 | 0.94 ± 0.1 | 0.80*** | 0.84 ± 0.09 | 0.94 ± 0.1 | 0.80*** |
| Middle/Inferior Temp | 0.99 ± 0.11 | 1.14 ± 0.13 | 0.81*** | 1.01 ± 0.11 | 1.16 ± 0.13 | 0.78*** |
| Fusiform | 0.90 ± 0.12 | 1.01 ± 0.13 | 0.84*** | 0.88 ± 0.12 | 0.99 ± 0.13 | 0.83*** |
| Posterior Temp | 1.06 ± 0.09 | 1.21 ± 0.11 | 0.78*** | 1.08 ± 0.09 | 1.23 ± 0.1 | 0.69** |
| Superior Temp (A) | 0.89 ± 0.1 | 1.00 ± 0.1 | 0.82*** | 0.91 ± 0.1 | 1.02 ± 0.11 | 0.80*** |
| Superior Temp (P) | 1.11 ± 0.09 | 1.27 ± 0.1 | 0.71** | 1.11 ± 0.09 | 1.28 ± 0.1 | 0.68** |
| *Insula and Cingulate* |  |  |  |  |  |  |
| PLG (insula) | 1.07 ± 0.1 | 1.14 ± 0.09 | 0.75** | 1.08 ± 0.09 | 1.15 ± 0.09 | 0.76** |
| ALG (insula) | 1.11 ± 0.1 | 1.22 ± 0.09 | 0.76*** | 1.11 ± 0.1 | 1.23 ± 0.09 | 0.71** |
| ASG (insula) | 1.17 ± 0.12 | 1.32 ± 0.12 | 0.72** | 1.17 ± 0.11 | 1.33 ± 0.12 | 0.75*** |
| MSG (insula) | 1.20 ± 0.11 | 1.32 ± 0.1 | 0.68** | 1.22 ± 0.11 | 1.33 ± 0.1 | 0.69** |
| PSG (insula) | 1.15 ± 0.1 | 1.26 ± 0.1 | 0.73** | 1.16 ± 0.1 | 1.27 ± 0.1 | 0.70** |
| AIC (insula) | 1.03 ± 0.11 | 1.11 ± 0.1 | 0.75*** | 1.06 ± 0.11 | 1.14 ± 0.11 | 0.81*** |
| ACC | 1.03 ± 0.1 | 1.15 ± 0.11 | 0.77*** | 1.03 ± 0.1 | 1.16 ± 0.11 | 0.79*** |
| PCC | 1.18 ± 0.11 | 1.19 ± 0.1 | 0.70** | 1.19 ± 0.1 | 1.41 ± 0.14 | 0.70** |
| *Frontal Lobe* |  |  |  |  |  |  |
| Middle Frontal | 1.18 ± 0.1 | 1.43 ± 0.13 | 0.61** | 1.19 ± 0.1 | 1.44 ± 0.14 | 0.64** |
| Precentral gyrus | 1.13 ± 0.09 | 1.33 ± 0.09 | 0.66** | 1.13 ± 0.09 | 1.32 ± 0.10 | 0.69** |
| Straight | 0.96 ± 0.11 | 1.13 ± 0.12 | 0.77*** | 1.17 ± 0.11 | 1.17 ± 0.12 | 0.78*** |
| Anterior Orbital | 1.12 ± 0.11 | 1.34 ± 0.13 | 0.65** | 1.12 ± 0.1 | 1.35 ± 0.13 | 0.71** |
| Inferior Frontal | 1.13 ± 0.1 | 1.36 ± 0.11 | 0.62** | 1.16 ± 0.09 | 1.38 ± 0.11 | 0.63** |
| Superior Frontal | 1.09 ± 0.1 | 1.29 ± 0.1 | 0.66** | 1.09 ± 0.1 | 1.30 ± 0.11 | 0.72*** |
| Medial Orbital | 1.01 ± 0.1 | 1.21 ± 0.11 | 0.73*** | 0.99 ± 0.1 | 1.19 ± 0.12 | 0.77*** |
| Lateral Orbital | 1.06 ± 0.12 | 1.29 ± 0.15 | 0.74*** | 1.09 ± 0.12 | 1.33 ± 0.15 | 0.72** |
| Posterior Orbital | 1.04 ± 0.11 | 1.20 ± 0.12 | 0.77*** | 1.04 ± 0.12 | 1.19 ± 0.13 | 0.81*** |
| Subgenual Frontal | 1.03 ± 0.11 | 1.11 ± 0.11 | 0.79*** | 1.00 ± 0.12 | 1.08 ± 0.12 | 0.85*** |
| Subcallosal | 0.91 ± 0.12 | 0.98 ± 0.12 | 0.82*** | 0.88 ± 0.13 | 0.95 ± 0.13 | 0.89*** |
| Pre-subgenual | 1.12 ± 0.14 | 1.23 ± 0.13 | 0.80*** | 1.09 ± 0.14 | 1.18 ± 0.14 | 0.84*** |
| *Occipital Lobe* |  |  |  |  |  |  |
| Lingual gyrus | 1.22 ± 0.1 | 1.46 ± 0.12 | 0.67** | 1.23 ± 0.11 | 1.46 ± 0.12 | 0.69** |
| Cuneus | 1.26 ± 0.11 | 1.52 ± 0.13 | 0.64** | 1.30 ± 0.11 | 1.57 ± 0.12 | 0.62** |
| Lateral occipital | 1.08 ± 0.12 | 1.30 ± 0.13 | 0.79*** | 1.10 ± 0.11 | 1.31 ± 0.13 | 0.72*** |
| *Parietal Lobe* |  |  |  |  |  |  |
| Postcentral gyrus | 1.09 ± 0.09 | 1.28 ± 0.09 | 0.64** | 1.10 ± 0.08 | 1.30 ± 0.1 | 0.66** |
| Superior Parietal | 1.15 + 0.1 | 1.39 ± 0.12 | 0.69** | 1.15 ± 0.1 | 1.37 ± 0.12 | 0.71** |
| Inferolateral Remainder | 1.09 ± 0.1 | 1.32 ± 0.17 | 0.77*** | 1.11 ± 0.11 | 1.33 ± 0.16 | 0.75*** |
| *Central Structures* |  |  |  |  |  |  |
| Caudate | 0.86 ± 0.17 | 1.03 ± 0.17 | 0.90*** | 0.84 ± 0.17 | 1.01 ± 0.19 | 0.92*** |
| Nucleus Accumbens | 1.07 ± 0.12 | 1.15 ± 0.13 | 0.76*** | 1.02 ± 0.12 | 1.10 ± 0.13 | 0.85*** |
| Putamen | 1.38 ± 0.13 | 1.55 ± 0.13 | 0.65** | 1.36 ± 0.12 | 1.53 ± 0.12 | 0.63** |
| Thalamus | 1.03 ± 0.15 | 1.03 ± 0.14 | 0.93*** | 1.02 ± 0.17 | 1.02 ± 0.16 | 0.94*** |
| Pallidum | 1.28 ± 0.14 | 1.37 ± 0.15 | 0.66** | 1.31 ± 0.13 | 1.46 ± 0.17 | 0.69** |
| Composite ROI | 1.09 ± 0.09 | 1.28 ± 0.13 | 0.74** | 1.10 ± 0.09 | 1.30 ± 0.13 | 0.74** |

** p-value < 0.001, *** p-value < 2e-16

**Supplementary Table 3: Bland-Altman Plot Statistics (EF and FDG)**

|  | Left |  |  | Right |  |  | |
| --- | --- | --- | --- | --- | --- | --- | --- |
| ROI | Avg. of differences | SD of differences | Lower CI, Upper CI | Avg. of differences | SD of differences | Lower CI,  Upper CI |  |
| *Temporal Lobe* |  |  |  |  |  |  |  |
| Hippocampus | 0.045 | 0.063 | -0.08, 0.17 | 0.049 | 0.061 | -0.07, 0.17 |  |
| Amygdala | 0.026 | 0.069 | -0.11, 0.16 | 0.031 | 0.063 | -0.09, 0.15 |  |
| Anterior Temporal (M) | 0.081 | 0.063 | -0.04, 0.21 | 0.078 | 0.057 | -0.03, 0.19 |  |
| Anterior Temporal (L) | 0.119 | 0.072 | -0.02, 0.26 | 0.115 | 0.078 | -0.04, 0.27 |  |
| Parahippocampal | 0.091 | 0.059 | -0.03, 0.21 | 0.097 | 0.059 | -0.02, 0.21 |  |
| Middle/Inferior Temp | 0.15 | 0.08 | 0.00, 0.31 | 0.15 | 0.08 | -0.02, 0.31 |  |
| Fusiform | 0.11 | 0.07 | -0.03, 0.24 | 0.11 | 0.07 | -0.03, 0.26 |  |
| Posterior Temp | 0.15 | 0.07 | 0.02, 0.29 | 0.15 | 0.08 | 0.00, 0.30 |  |
| Superior Temp (A) | 0.11 | 0.06 | 0.00, 0.23 | 0.11 | 0.07 | -0.02, 0.24 |  |
| Superior Temp (P) | 0.16 | 0.07 | 0.03, 0.30 | 0.16 | 0.08 | 0.01, 0.31 |  |
| *Insula and Cingulate* |  |  |  |  |  |  |  |
| PLG (insula) | 0.07 | 0.07 | -0.06, 0.20 | 0.07 | 0.06 | -0.05, 0.20 |  |
| ALG (insula) | 0.11 | 0.07 | -0.02, 0.24 | 0.12 | 0.07 | -0.03, 0.26 |  |
| ASG (insula) | 0.16 | 0.09 | -0.02, 0.33 | 0.15 | 0.08 | -0.01, 0.31 |  |
| MSG (insula) | 0.12 | 0.08 | -0.04, 0.28 | 0.11 | 0.08 | -0.05, 0.27 |  |
| PSG (insula) | 0.11 | 0.07 | -0.03, 0.26 | 0.11 | 0.08 | -0.04, 0.27 |  |
| AIC (insula) | 0.09 | 0.07 | -0.06, 0.23 | 0.08 | 0.07 | -0.05, 0.21 |  |
| ACC | 0.12 | 0.07 | -0.02, 0.26 | 0.12 | 0.07 | -0.01, 0.26 |  |
| PCC | 0.21 | 0.1 | 0.02, 0.40 | 0.22 | 0.10 | 0.03, 0.41 |  |
| *Frontal Lobe* |  |  |  |  |  |  |  |
| Middle Frontal | 0.25 | 0.11 | 0.04, 0.46 | 0.24 | 0.10 | 0.04, 0.45 |  |
| Precentral gyrus | 0.20 | 0.08 | 0.05, 0.35 | 0.19 | 0.08 | 0.05, 0.34 |  |
| Straight | 0.17 | 0.08 | 0.02, 0.32 | 0.17 | 0.08 | 0.01, 0.32 |  |
| Anterior Orbital | 0.22 | 0.10 | 0.03, 0.42 | 0.23 | 0.09 | 0.06, 0.41 |  |
| Inferior Frontal | 0.23 | 0.09 | 0.05, 0.41 | 0.22 | 0.09 | 0.04, 0.39 |  |
| Superior Frontal | 0.21 | 0.08 | 0.05, 0.37 | 0.20 | 0.08 | 0.05, 0.36 |  |
| Medial Orbital | 0.20 | 0.08 | 0.05, 0.36 | 0.20 | 0.08 | 0.05, 0.35 |  |
| Lateral Orbital | 0.23 | 0.10 | 0.04, 0.43 | 0.23 | 0.10 | 0.03, 0.43 |  |
| Posterior Orbital | 0.16 | 0.08 | 0.12, 0.31 | 0.15 | 0.07 | 0.01, 0.30 |  |
| Subgenual Frontal | 0.09 | 0.07 | -0.06, 0.23 | 0.08 | 0.07 | -0.05, 0.21 |  |
| Subcallosal | 0.08 | 0.07 | -0.06, 0.22 | 0.07 | 0.06 | -0.06, 0.20 |  |
| Pre-subgenual | 0.11 | 0.08 | -0.05, 0.27 | 0.10 | 0.08 | -0.06, 0.25 |  |
| *Occipital Lobe* |  |  |  |  |  |  |  |
| Lingual gyrus | 0.23 | 0.09 | 0.05, 0.41 | 0.24 | 0.10 | 0.06, 0.41 |  |
| Cuneus | 0.26 | 0.10 | 0.07, 0.46 | 0.27 | 0.10 | 0.07, 0.47 |  |
| Lateral occipital | 0.22 | 0.08 | 0.05, 0.38 | 0.21 | 0.09 | 0.04, 0.39 |  |
| *Parietal Lobe* |  |  |  |  |  |  |  |
| Postcentral gyrus | 0.19 | 0.08 | 0.05, 0.34 | 0.19 | 0.08 | 0.04, 0.35 |  |
| Superior Parietal | 0.24 | 0.09 | 0.07, 0.41 | 0.23 | 0.09 | 0.06, 0.40 |  |
| Inferolateral Remainder | 0.23 | 0.11 | 0.01, 0.44 | 0.21 | 0.10 | 0.01, 0.41 |  |
| *Central Structures* |  |  |  |  |  |  |  |
| Caudate | 0.16 | 0.08 | 0.01, 0.31 | 0.17 | 0.07 | 0.03, 0.31 |  |
| Nucleus Accumbens | 0.08 | 0.08 | -0.08, 0.25 | 0.08 | 0.07 | -0.06, 0.22 |  |
| Putamen | 0.17 | 0.10 | -0.03, 0.38 | 0.18 | 0.10 | -0.02, 0.37 |  |
| Thalamus | 0.00 | 0.06 | -0.11, 0.11 | 0.00 | 0.06 | -0.12, 0.11 |  |
| Pallidum | 0.09 | 0.12 | -0.14, 0.33 | 0.15 | 0.13 | -0.10, 0.40 |  |
| Composite ROI | 0.20 | 0.09 | 0.03, 0.36 | 0.19 | 0.09 | 0.02, 0.37 |  |

**Supplementary Table 4: Interactions of Disease State and FDG in Explaining early-frame Amyloid**

|  | Left |  | Right |  |
| --- | --- | --- | --- | --- |
| ROI | Dementia x FDG | MCI x FDG | Dementia x FDG | MCI x FDG |
| *Temporal Lobe* |  |  |  |  |
| Hippocampus | -0.02 | 0.15 | -0.14 | **0.35*** |
| Amygdala | 0.07 | 0.29 | 0.19 | 0.42 |
| Anterior Temporal (M) | 0.01 | 0.14 | -0.07 | 0.16 |
| Anterior Temporal (L) | -0.14 | 0.14 | -0.25 | 0.001 |
| Parahippocampal | -0.26 | 0.06 | -0.02 | 0.09 |
| Middle/Inferior Temp | -0.22 | 0.08 | -0.07 | -0.04 |
| Fusiform | -0.24 | 0.12 | -0.22 | -0.11 |
| Posterior Temp | -0.09 | 0.01 | 0.05 | -0.16 |
| Superior Temp (A) | -0.13 | 0.29 | 0.11 | 0.15 |
| Superior Temp (P) | 0.03 | 0.21 | 0.30 | 0.23 |
| *Insula and Cingulate* |  |  |  |  |
| PLG (insula) | -0.14 | 0.12 | -0.07 | 0.25 |
| ALG (insula) | -0.10 | 0.12 | -0.34 | 0.31 |
| ASG (insula) | -0.15 | 0.17 | -0.05 | 0.29 |
| MSG (insula) | -0.04 | 0.14 | -0.18 | 0.40 |
| PSG (insula) | -0.03 | 0.16 | -0.19 | 0.16 |
| AIC (insula) | -0.29 | -0.03 | 0.11 | 0.12 |
| ACC | -0.02 | 0.24 | 0.28 | 0.29 |
| PCC | 0.06 | 0.03 | -0.04 | 0.04 |
| *Frontal Lobe* |  |  |  |  |
| Middle Frontal | 0.002 | 0.16 | 0.04 | 0.14 |
| Precentral gyrus | -0.04 | 0.36 | 0.04 | 0.22 |
| Straight | 0.04 | 0.10 | -0.03 | 0.02 |
| Anterior Orbital | -0.12 | 0.07 | -0.02 | -0.01 |
| Inferior Frontal | -0.13 | 0.18 | -0.08 | 0.10 |
| Superior Frontal | 0.01 | 0.33 | -0.04 | 0.23 |
| Medial Orbital | -0.21 | 0.03 | -0.15 | 0.02 |
| Lateral Orbital | -0.08 | 0.08 | -0.14 | -0.19 |
| Posterior Orbital | 0.001 | 0.05 | -0.01 | -0.17 |
| Subgenual Frontal | 0.15 | 0.39 | 0.12 | 0.39 |
| Subcallosal | 0.31 | 0.24 | 0.05 | 0.13 |
| Pre-subgenual | 0.33 | 0.37 | 0.15 | 0.24 |
| *Occipital Lobe* |  |  |  |  |
| Lingual gyrus | 0.07 | 0.12 | 0.04 | -0.01 |
| Cuneus | -0.13 | -0.10 | -0.04 | 0.13 |
| Lateral occipital | -0.08 | -0.05 | -0.08 | -0.15 |
| *Parietal Lobe* |  |  |  |  |
| Postcentral gyrus | -0.003 | 0.31 | 0.11 | 0.23 |
| Superior Parietal | 0.06 | 0.07 | 0.10 | 0.06 |
| Inferolateral Remainder | 0.04 | 0.05 | 0.10 | -0.05 |
| *Central Structures* |  |  |  |  |
| Caudate | 0.15 | 0.15 | 0.31 | 0.20 |
| Nucleus Accumbens | 0.24 | 0.11 | 0.02 | 0.08 |
| Putamen | -0.12 | 0.23 | -0.25 | 0.18 |
| Thalamus | 0.25 | 0.21 | **0.37*** | **0.37*** |
| Pallidum | -0.01 | 0.06 | 0.23 | 0.31 |
| Composite ROI | -0.01 | 0.05 | -0.09 | -0.01 |

* denotes a significant adjusted p-value (p<0.05)

**Supplementary Table 5: Linear Regression for Early-frame Florbetapir and Early-frame Florbetaben Comparison** Covariates include age, diagnostic group (CU vs MCI), and CDR sum of boxes score, FDR corrected; Estimate of tracer: Florbetaben compared to Florbetapir

|  | Left |  |  |  | Right |  |  |  |
| --- | --- | --- | --- | --- | --- | --- | --- | --- |
| ROI | FBP  Mean (SD) | FBB  Mean (SD) | Estimate of tracer | Adjusted P-value | FBP  Mean (SD) | FBB  Mean (SD) | Estimate of tracer | Adjusted P-value |
| *Temporal Lobe* |  |  |  |  |  |  |  |  |
| Hippocampus | 0.97 (0.09) | 0.94 (0.09) | -0.04 | 0.24 | 0.97 (0.09) | 0.91 (0.10) | -0.06 | 0.10 |
| Amygdala | 0.94 (0.10) | 0.90 (0.11) | -0.05 | 0.24 | 0.92 (0.10) | 0.86 (0.11) | -0.06 | 0.11 |
| Anterior Temporal (M) | 0.86 (0.10) | 0.85 (0.09) | -0.001 | 0.96 | 0.86 (0.10) | 0.85 (0.10) | -0.01 | 0.89 |
| Anterior Temporal (L) | 0.98 (0.12) | 0.96 (0.13) | -0.02 | 0.64 | 0.99 (0.12) | 0.97 (0.10) | -0.02 | 0.73 |
| Parahippocampal | 1.00 (0.09) | 0.89 (0.09) | 0.01 | 0.89 | 0.88 (0.10) | 0.88 (0.08) | -0.003 | 0.96 |
| Middle/Inferior Temp | 1.03 (0.11) | 1.05 (0.11) | 0.01 | 0.74 | 1.05 (0.11) | 1.06 (0.08) | 0.01 | 0.88 |
| Fusiform | 0.95 (0.12) | 0.97 (0.12) | 0.02 | 0.70 | 0.93 (0.11) | 0.94 (0.11) | 0.01 | 0.74 |
| Posterior Temp | 1.08 (0.09) | 1.12 (0.08) | 0.04 | 0.26 | 1.10 (0.09) | 1.13 (0.08) | 0.02 | 0.44 |
| Superior Temp (A) | 0.90 (0.09) | 0.87 (0.09) | -0.03 | 0.36 | 0.94 (0.09) | 0.90 (0.10) | -0.03 | 0.43 |
| Superior Temp (P) | 1.13 (0.09) | 1.15 (0.09) | 0.02 | 0.57 | 1.14 (0.08) | 1.15 (0.09) | 0.001 | 0.96 |
| *Insula and Cingulate* |  |  |  |  |  |  |  |  |
| PLG (insula) | 1.08 (0.10) | 1.09 (0.09) | -0.001 | 0.96 | 1.10 (0.09) | 1.09 (0.09) | -0.02 | 0.63 |
| ALG (insula) | 1.12 (0.10) | 1.13 (0.10) | -0.003 | 0.96 | 1.13 (0.10) | 1.14 (0.11) | -0.0003 | 0.96 |
| ASG (insula) | 1.19 (0.11) | 1.15 (0.12) | -0.05 | 0.24 | 1.20 (0.10) | 1.15 (0.13) | -0.06 | 0.19 |
| MSG (insula) | 1.21 (0.11) | 1.20 (0.11) | -0.02 | 0.67 | 1.23 (0.10) | 1.19 (0.11) | -0.04 | 0.27 |
| PSG (insula) | 1.16 (0.10) | 1.16 (0.10) | -0.003 | 0.96 | 1.17 (0.10) | 1.17 (0.10) | -0.01 | 0.74 |
| AIC (insula) | 1.04 (0.11) | 1.07 (0.12) | 0.02 | 0.63 | 1.08 (0.11) | 1.06 (0.14) | -0.02 | 0.64 |
| ACC | 1.06 (0.11) | 1.06 (0.14) | -0.002 | 0.96 | 1.06 (0.10) | 1.08 (0.13) | 0.01 | 0.74 |
| PCC | 1.21 (0.12) | 1.26 (0.02) | 0.04 | 0.37 | 1.22 (0.11) | 1.27 (0.15) | 0.04 | 0.37 |
| *Frontal Lobe* |  |  |  |  |  |  |  |  |
| Middle Frontal | 1.18 (0.11) | 1.21 (0.10) | 0.03 | 0.43 | 1.20 (0.10) | 1.22 (0.12) | 0.02 | 0.63 |
| Precentral gyrus | 1.13 (0.10) | 1.16 (0.12) | 0.03 | 0.37 | 1.13 (0.10) | 1.17 (0.12) | 0.04 | 0.36 |
| Straight | 0.98 (0.12) | 1.02 (0.10) | 0.03 | 0.43 | 1.02 (0.12) | 1.05 (0.10) | 0.03 | 0.43 |
| Anterior Orbital | 1.11 (0.12) | 1.14 (0.11) | 0.03 | 0.57 | 1.11 (0.12) | 1.14 (0.13) | 0.03 | 0.50 |
| Inferior Frontal | 1.15 (0.11) | 1.19 (0.12) | 0.04 | 0.36 | 1.18 (0.10) | 1.20 (0.12) | 0.02 | 0.63 |
| Superior Frontal | 1.09 (0.10) | 1.11 (0.10) | 0.02 | 0.63 | 1.09 (0.11) | 1.13 (0.10) | 0.03 | 0.37 |
| Medial Orbital | 1.10 (0.11) | 1.06 (0.10) | 0.05 | 0.24 | 1.00 (0.12) | 1.05 (0.11) | 0.06 | 0.19 |
| Lateral Orbital | 1.06 (0.13) | 1.13 (0.14) | 0.07 | 0.20 | 1.10 (0.12) | 1.15 (0.13) | 0.05 | 0.24 |
| Posterior Orbital | 1.04 (0.11) | 1.06 (0.10) | 0.03 | 0.49 | 1.04 (0.12) | 1.07 (0.12) | 0.03 | 0.50 |
| Subgenual Frontal | 1.04 (0.11) | 0.96 (0.20) | -0.08 | 0.11 | 1.00 (0.11) | 0.92 (0.21) | -0.09 | 0.10 |
| Subcallosal | 0.94 (0.11) | 0.86 (0.14) | -0.08 | 0.10 | 1.00 (0.11) | 0.83 (0.16) | -0.08 | 0.10 |
| Pre-subgenual | 1.12 (0.13) | 1.05 (0.13) | -0.08 | 0.11 | 1.08 (0.13) | 1.02 (0.16) | -0.07 | 0.19 |
| *Occipital Lobe* |  |  |  |  |  |  |  |  |
| Lingual gyrus | 1.23 (0.10) | 1.31 (0.11) | 0.08 | 0.10 | 1.24 (0.11) | 1.29 (0.13) | 0.05 | 0.24 |
| Cuneus | 1.27 (0.12) | 1.36 (0.10) | 0.08 | 0.10 | 1.31 (0.12) | 1.38 (0.11) | 0.07 | 0.11 |
| Lateral occipital | **1.08 (0.12)** | **1.19 (0.11)** | **0.103** | **0.04** | 1.11 (0.12) | 1.21 (0.12) | 0.09 | 0.08 |
| *Parietal Lobe* |  |  |  |  |  |  |  |  |
| Postcentral gyrus | 1.10 (0.09) | 1.14 (0.12) | 0.04 | 0.24 | 1.11 (0.09) | 1.14 (0.12) | 0.03 | 0.37 |
| Superior Parietal | 1.17 (0.11) | 1.22 (0.11) | 0.05 | 0.24 | 1.16 (0.10) | 1.21 (0.11) | 0.05 | 0.24 |
| Inferolateral Remainder | 1.12 (0.10) | 1.18 (0.10) | 0.06 | 0.11 | 1.14 (0.10) | 1.18 (0.10) | 0.04 | 0.30 |
| *Central Structures* |  |  |  |  |  |  |  |  |
| Caudate | 0.91 (0.16) | 0.83 (0.21) | -0.09 | 0.19 | 0.89 (0.15) | 0.83 (0.20) | -0.07 | 0.24 |
| Nucleus Accumbens | 1.08 (0.12) | 1.04 (0.10) | -0.04 | 0.32 | 1.05 (0.12) | 1.00 (0.10) | -0.05 | 0.24 |
| Putamen | 1.38 (0.13) | 1.37 (0.10) | -0.01 | 0.85 | 1.36 (0.12) | 1.35 (0.10) | -0.01 | 0.84 |
| Thalamus | 1.07 (0.14) | 1.01 (0.18) | -0.07 | 0.20 | 1.07 (0.13) | 1.04 (0.17) | -0.04 | 0.40 |
| Pallidum | 1.27 (0.13) | 1.19 (0.10) | -0.09 | 0.10 | 1.31 (0.14) | 1.26 (0.11) | -0.01 | 0.26 |
| Composite ROI | 1.12 (0.09) | 1.16 (0.10) | 0.04 | 0.27 | 1.14 (0.09) | 1.17 (0.09) | 0.03 | 0.39 |

**Supplementary Table 6: Anatomical Regions Corresponding to Figure 6**

| **Number** | **ROI** | **Number** | **ROI** |
| --- | --- | --- | --- |
| 1 | Right Hippocampus | 43 | Left Straight Gyrus |
| 2 | Left Hippocampus | 44 | Right Straight Gyrus |
| 3 | Right Amygdala | 45 | Left Anterior Orbital Gyrus |
| 4 | Left Amygdala | 46 | Right Anterior Orbital Gyrus |
| 5 | Right Anterior Temporal (medial) | 47 | Left Inferior Frontal Gyrus |
| 6 | Left Anterior Temporal (medial) | 48 | Right Inferior Frontal Gyrus |
| 7 | Right Anterior Temporal (lateral) | 49 | Left Superior Frontal Gyrus |
| 8 | Left Anterior Temporal (lateral) | 50 | Right Superior Frontal Gyrus |
| 9 | Right Parahippocampal | 51 | Left Postcentral Gyrus |
| 10 | Left Parahippocampal | 52 | Right Postcentral Gyrus |
| 11 | Right Superior Temporal (posterior) | 53 | Left Superior Parietal Gyrus |
| 12 | Left Superior Temporal (posterior) | 54 | Right Superior Parietal Gyrus |
| 13 | Right Middle/Inferior Temporal | 55 | Left Lingual Gyrus |
| 14 | Left Middle/Inferior Temporal | 56 | Right Lingual Gyrus |
| 15 | Right Fusiform Gyrus | 57 | Left Cuneus |
| 16 | Left Fusiform Gyrus | 58 | Right Cuneus |
| 17 | Left Posterior Long Gyrus (insula) | 59 | Left Medial Orbital Gyrus |
| 18 | Right Posterior Long Gyrus (insula) | 60 | Right Medial Orbital Gyrus |
| 19 | Left Lateral Occipital Lobe | 61 | Left Lateral Orbital Gyrus |
| 20 | Right Lateral Occipital Lobe | 62 | Right Lateral Orbital Gyrus |
| 21 | Left Anterior Cingulate | 63 | Left Posterior Orbital Gyrus |
| 22 | Right Anterior Cingulate | 64 | Right Posterior Orbital Gyrus |
| 23 | Left Posterior Cingulate | 65 | Left Subgenual Frontal Cortex |
| 24 | Right Posterior Cingulate | 66 | Right Subgenual Frontal Cortex |
| 25 | Left Middle Frontal Gyrus | 67 | Left Subcallosal Area |
| 26 | Right Middle Frontal Gyrus | 68 | Right Subcallosal Area |
| 27 | Left Posterior Temporal | 69 | Left Pre-subgenual Frontal Cortex |
| 28 | Right Posterior Temporal | 70 | Right Pre-subgenual Frontal Cortex |
| 29 | Left Inferiolateral Remainder of Parietal Lobe | 71 | Left Superior Temporal (anterior) |
| 30 | Right Inferiolateral Remainder of Parietal Lobe | 72 | Right Superior Temporal (anterior) |
| 31 | Left Caudate | 73 | Left Anterior Short Gyrus (insula) |
| 32 | Right Caudate | 74 | Right Anterior Short Gyrus (insula) |
| 33 | Left Nucleus Accumbens | 75 | Left Middle Short Gyrus (insula) |
| 34 | Right Nucleus Accumbens | 76 | Right Middle Short Gyrus (insula) |
| 35 | Left Putamen | 77 | Left Posterior Short Gyrus (insula) |
| 36 | Right Putamen | 78 | Right Posterior Short Gyrus (insula) |
| 37 | Left Thalamus | 79 | Left Anterior Inferior Cortex (insula) |
| 38 | Right Thalamus | 80 | Right Anterior Inferior Cortex (insula) |
| 39 | Left Pallidum | 81 | Left Anterior Long Gyrus (insula) |
| 40 | Right Pallidum | 82 | Right Anterior Long Gyrus (insula) |
| 41 | Left Precentral Gyrus | 83 | Left Composite Region |
| 42 | Right Precentral Gyrus | 84 | Right Composite Region |

**Supplementary Figure 1: Bland-Altman Plots**

**
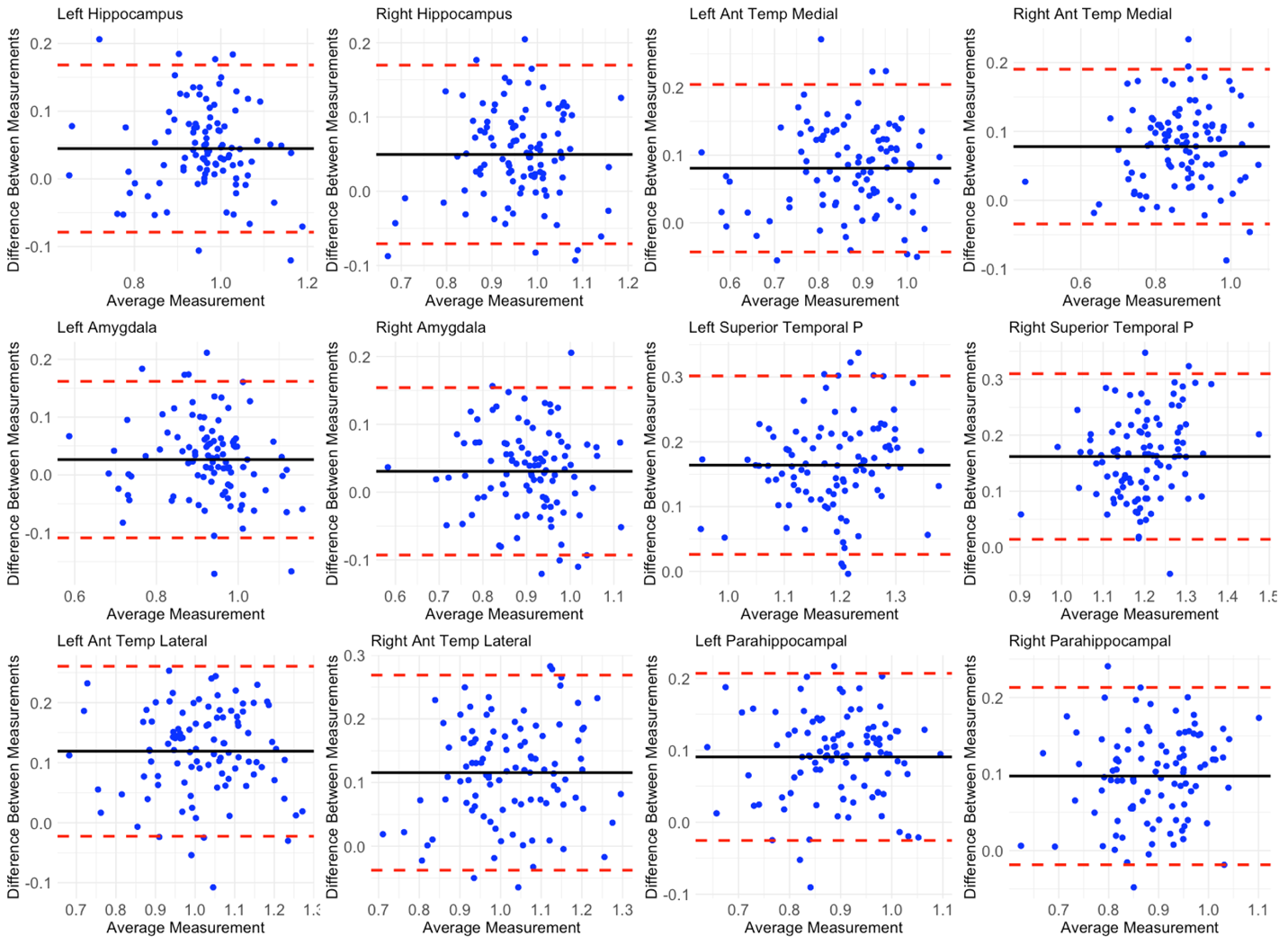
**

**
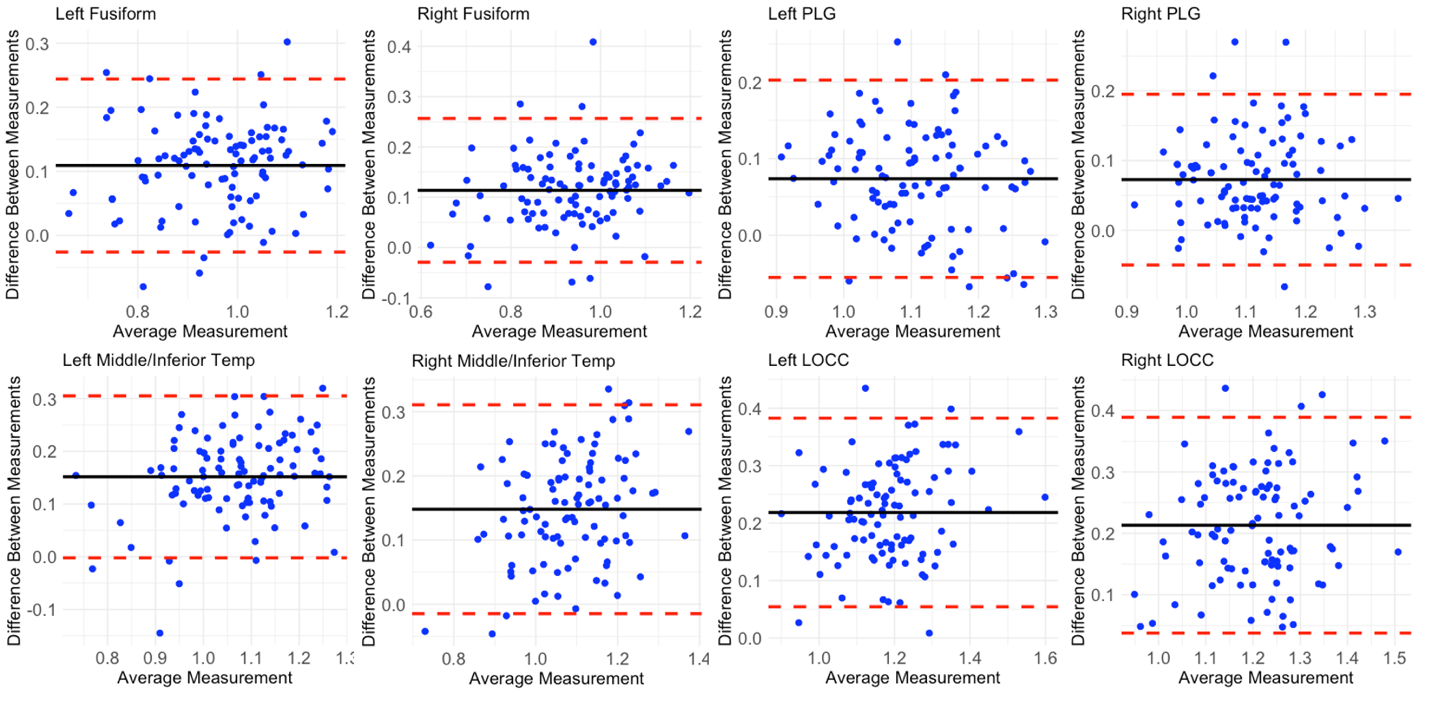
**

**
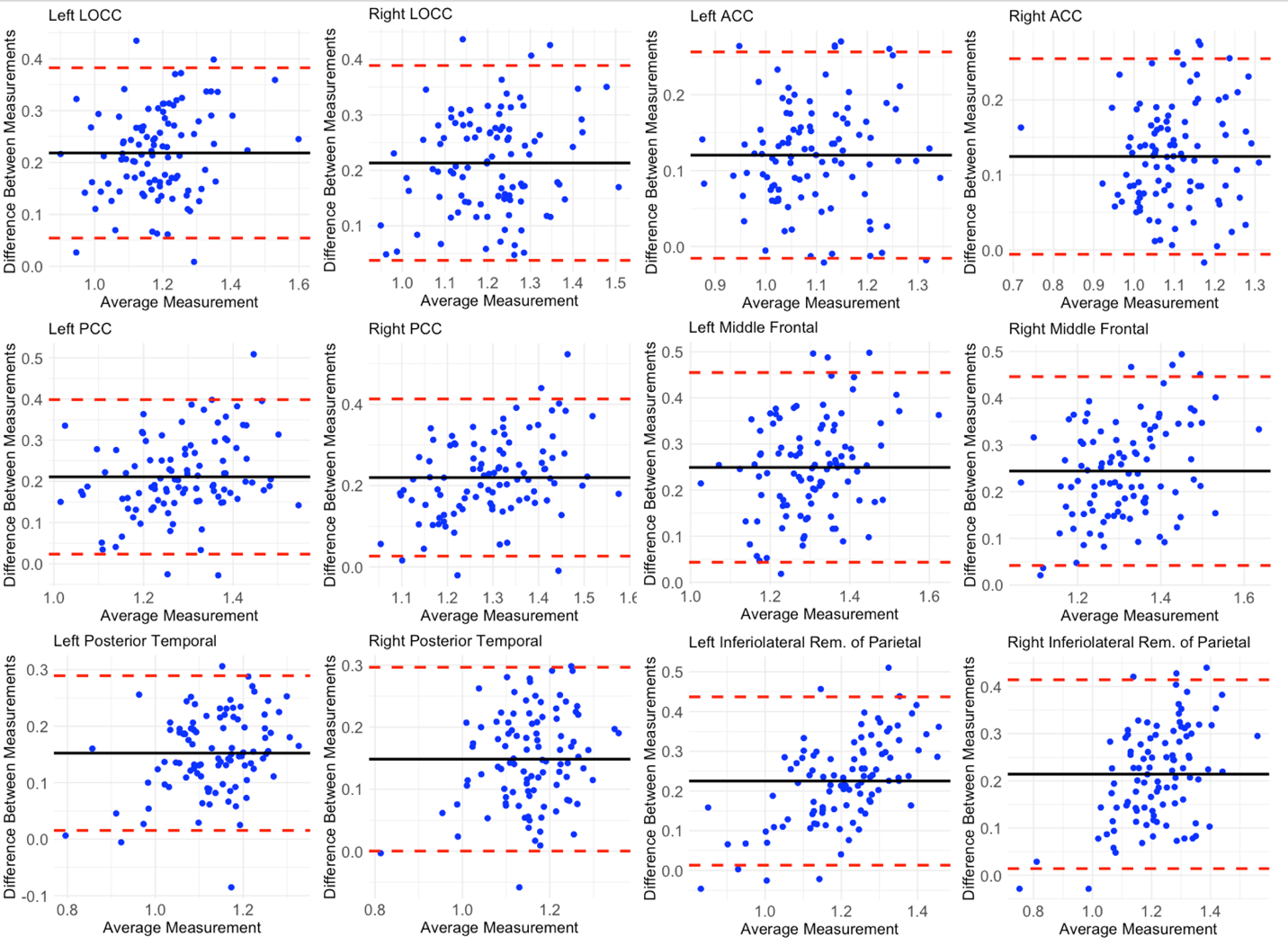
**

**
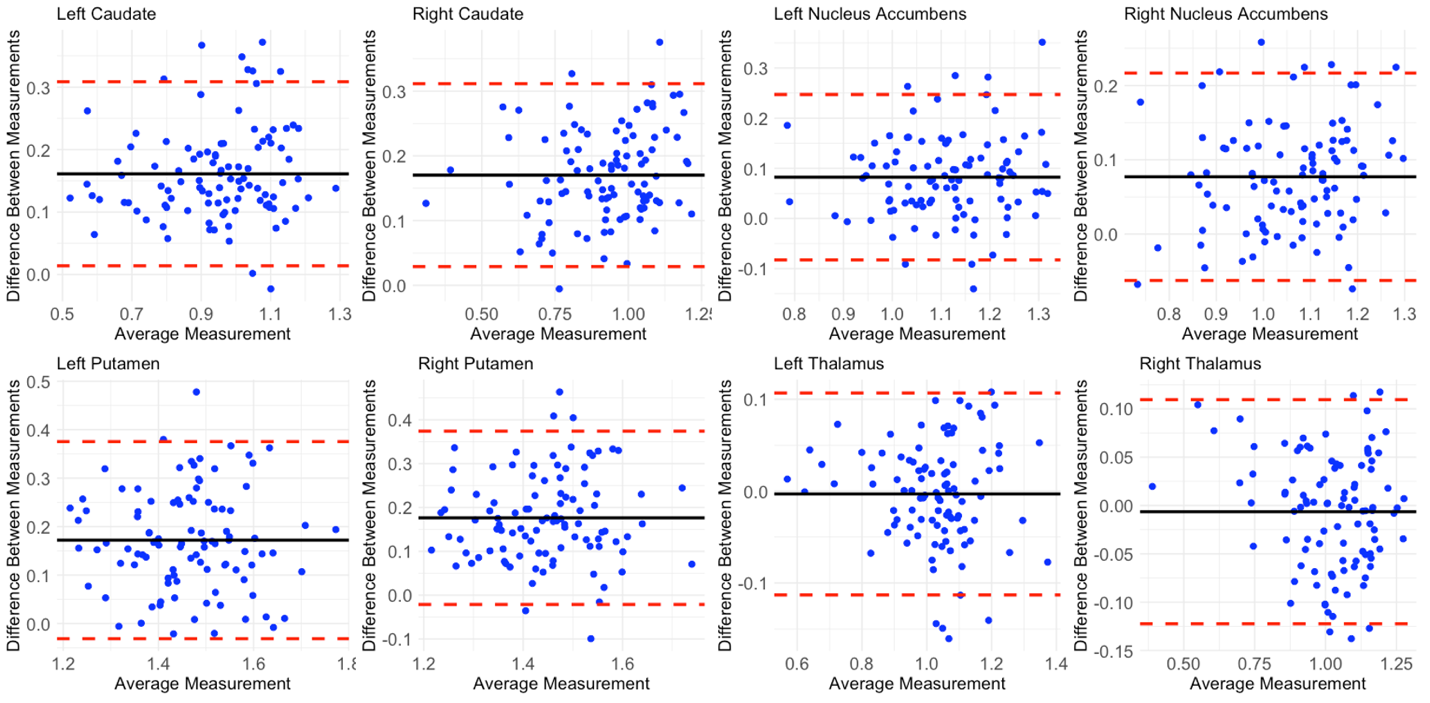
**

**
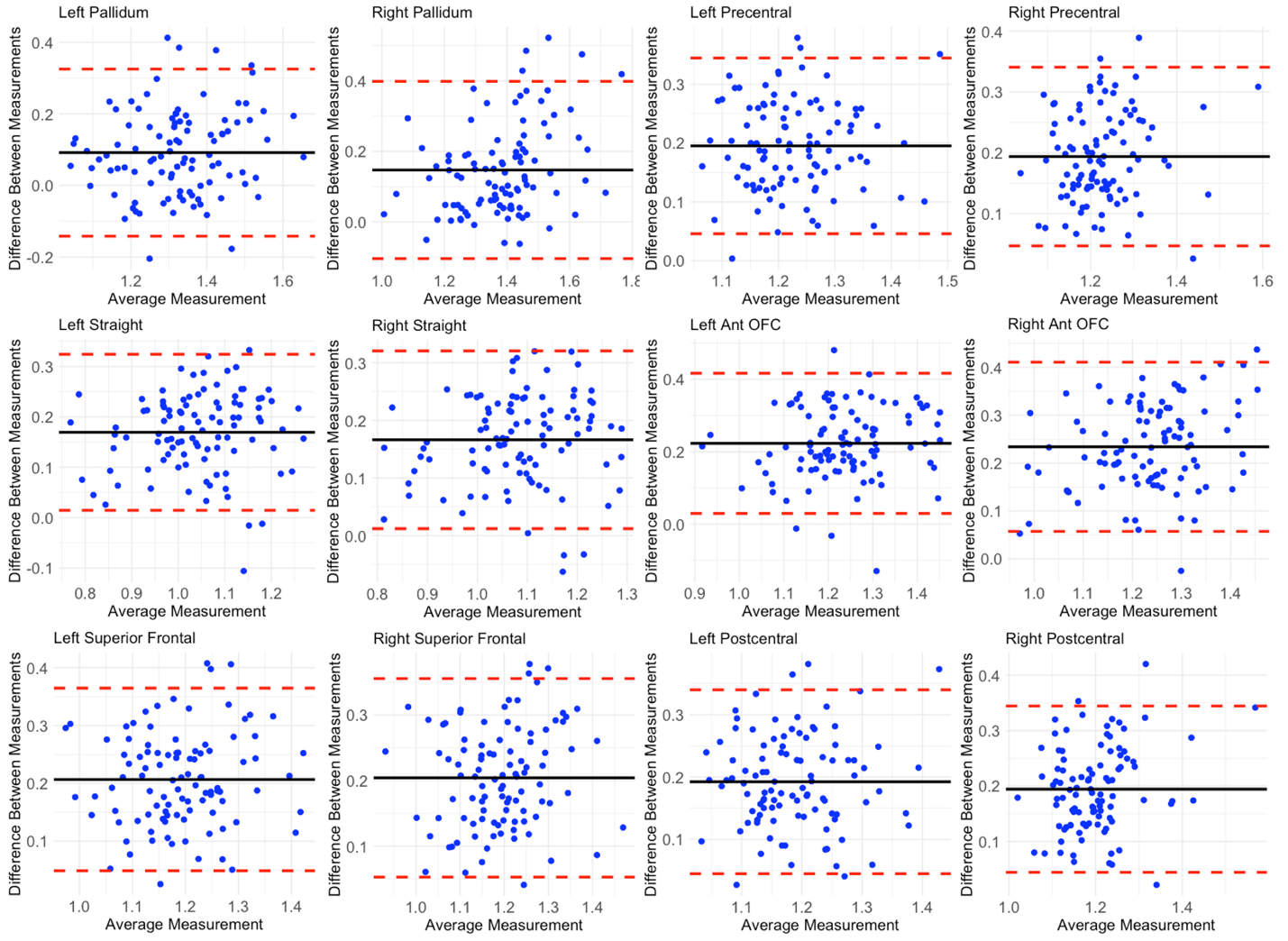
**

**
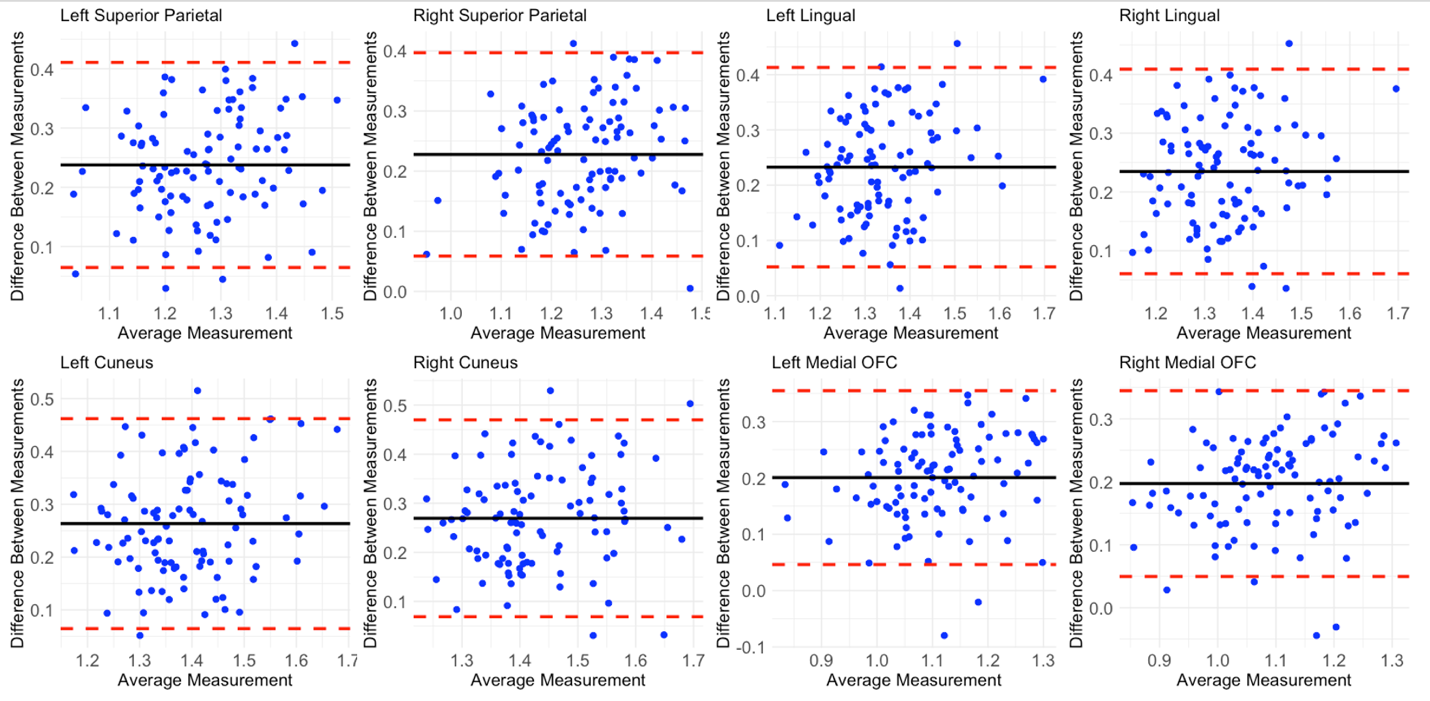
**

**
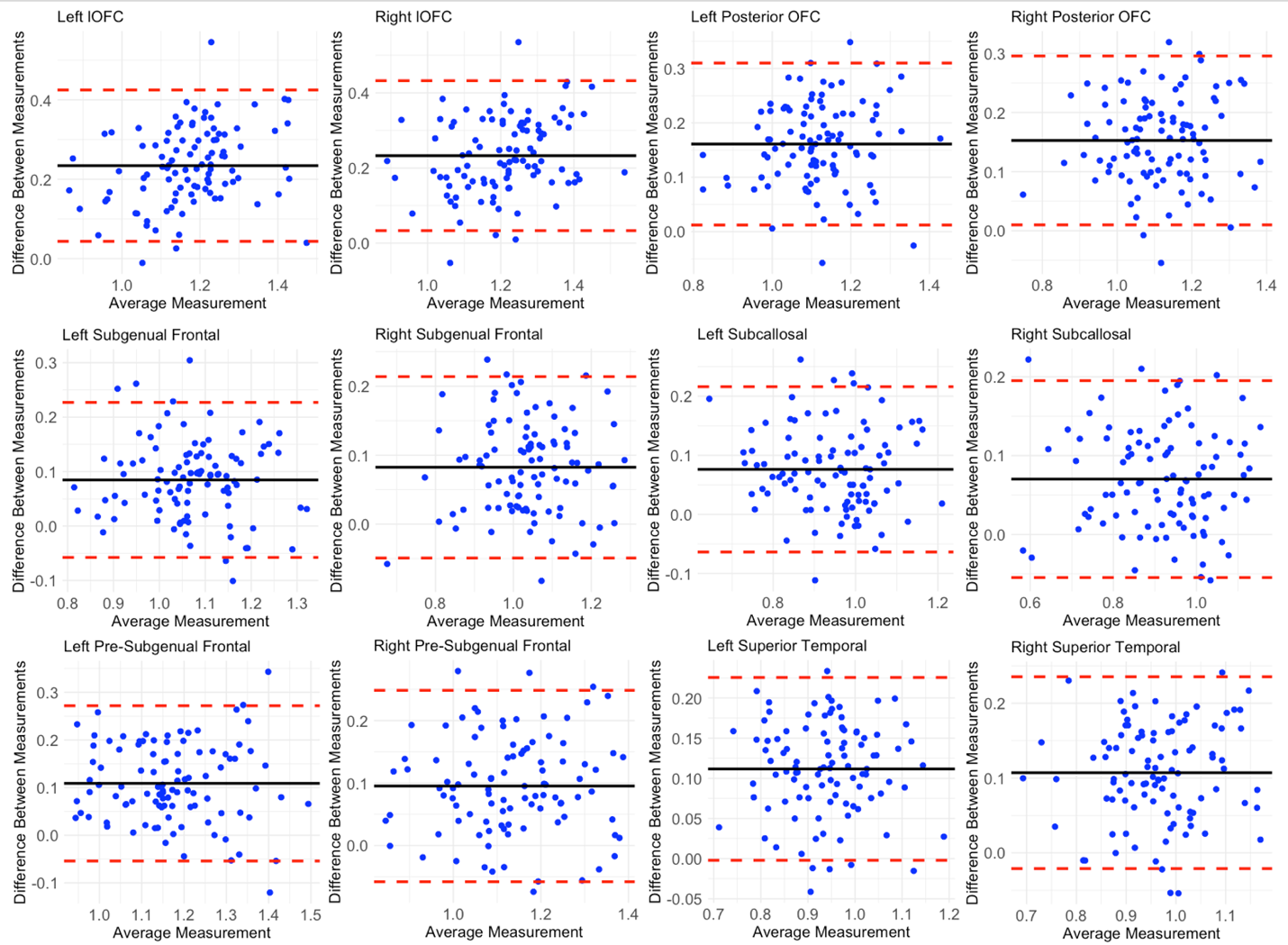
**

**
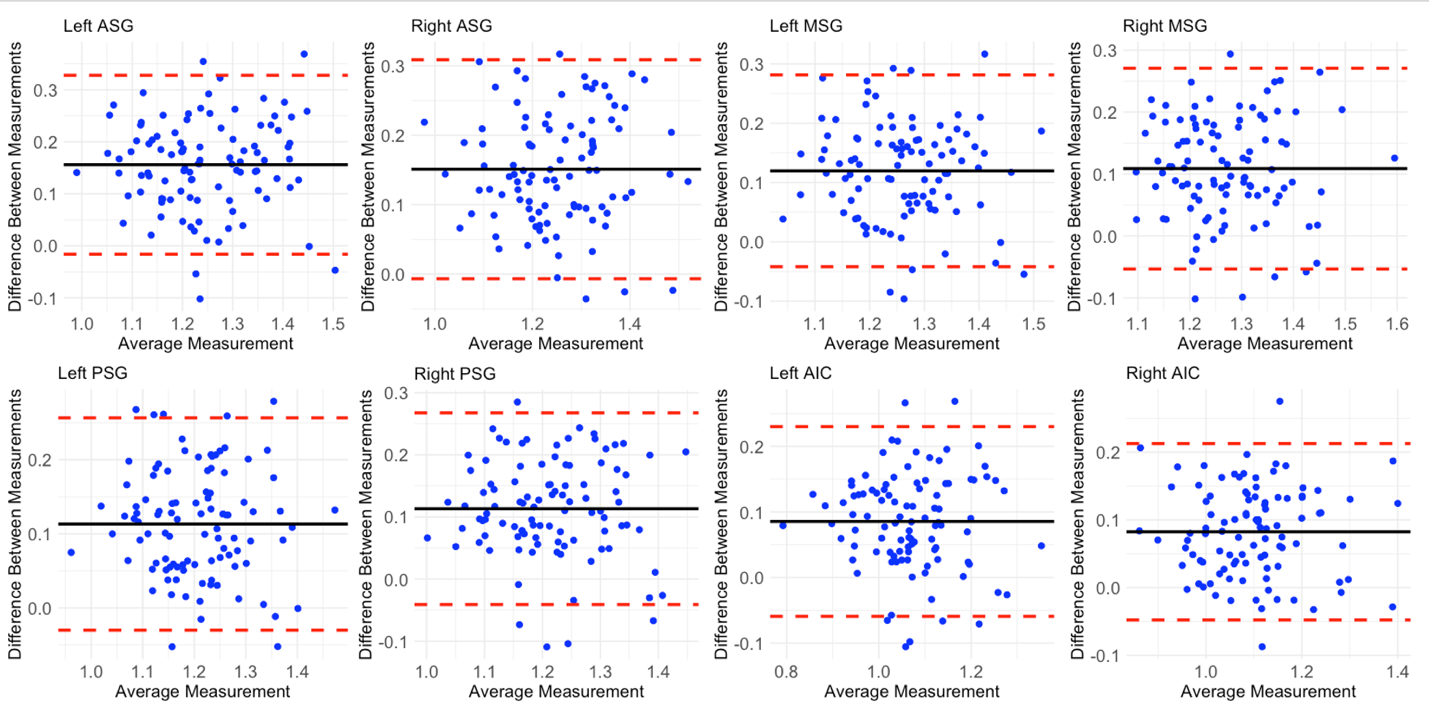
**

**Supplementary Figure 2: Histogram of Cosine Similarity Distribution**


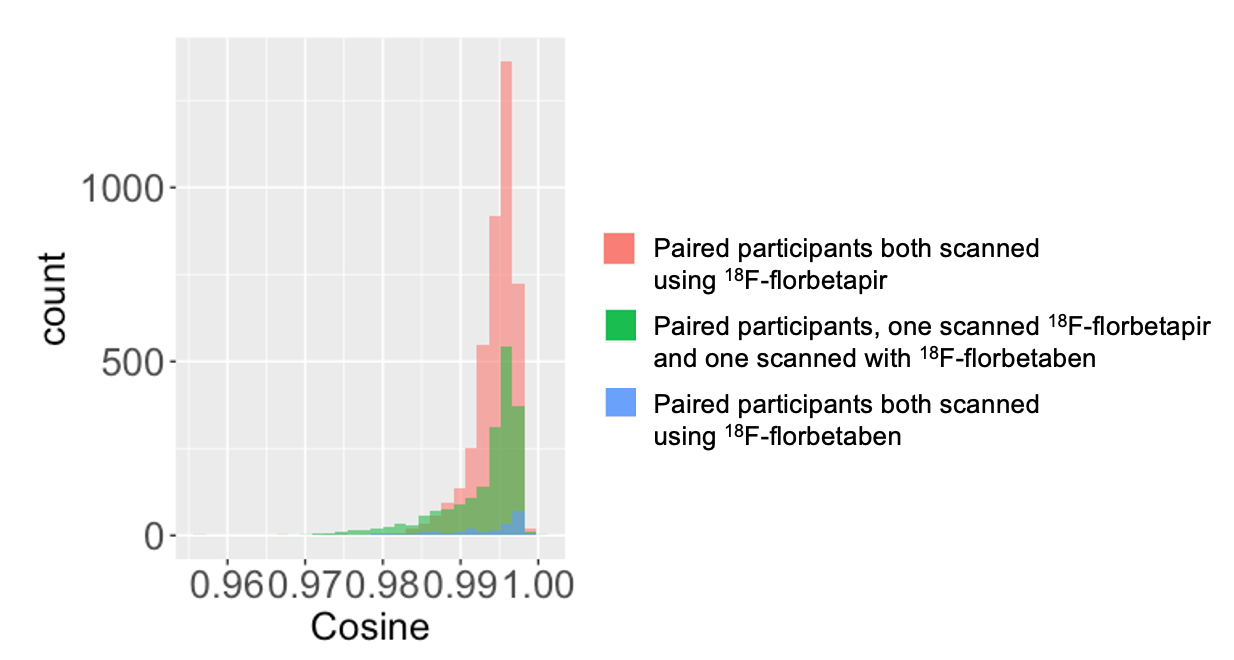

Supplement: Supplementary file 1 — Additional file 1. Supplementary Information. [file 41824_2021_123_MOESM1_ESM.docx]
